# Genetic sexing strains for the population suppression of the mosquito vector *Aedes aegypti*

**DOI:** 10.1098/rstb.2019.0808

**Published:** 2020-12-28

**Authors:** Panagiota Koskinioti, Antonios A. Augustinos, Danilo O. Carvalho, Muhammad Misbah-ul-Haq, Gulizar Pillwax, Lucia Duran de la Fuente, Gustavo Salvador-Herranz, Rafael Argilés Herrero, Kostas Bourtzis

**Affiliations:** 1 Insect Pest Control Laboratory, Joint FAO/IAEA Programme of Nuclear Techniques in Food and Agriculture, Seibersdorf, Vienna, Austria; 2 Department of Biochemistry and Biotechnology, University of Thessaly, Larissa, Greece; 3 Nuclear Institute for Food and Agriculture, Peshawar, Pakistan; 4 Departamento de Expresión Gráfica, Proyectos y Urbanismo, Universidad CEU Cardenal Herrera, Valencia, Spain; 5 Insect Pest Control Section, Joint FAO/IAEA Programme of Nuclear Techniques in Food and Agriculture, Wagramerstrasse 5, PO Box 100, 1400 Vienna, Austria

**Keywords:** sterile insect technique, vector control, dengue, Zika

## Abstract

*Aedes aegypti* is the primary vector of arthropod-borne viruses including dengue, chikungunya and Zika. Vector population control methods are reviving to impede disease transmission. An efficient sex separation for male-only releases is crucial for area-wide mosquito population suppression strategies. Here, we report on the construction of two genetic sexing strains using red- and white-eye colour mutations as selectable markers. Quality control analysis showed that the Red-eye genetic sexing strains (GSS) is better and more genetically stable than the White-eye GSS. The introduction of an irradiation-induced inversion (Inv35) increases genetic stability and reduces the probability of female contamination of the male release batches. Bi-weekly releases of irradiated males of both the Red-eye GSS and the Red-eye GSS/Inv35 fully suppressed target laboratory cage populations within six and nine weeks, respectively. An image analysis algorithm allowing sex determination based on eye colour identification at the pupal stage was developed. The next step is to automate the Red-eye-based genetic sexing and validate it in pilot trials prior to its integration in large-scale population suppression programmes.

This article is part of the theme issue ‘Novel control strategies for mosquito-borne diseases’.

## 1. Introduction

Over 3.5 billion people are at risk of contracting *Aedes*-transmitted diseases including dengue, chikungunya, Zika and yellow fever [1,2]. Conventional vector control programmes, largely based on the removal of breeding sites and the application of insecticides, have been unable to sustainably reduce the populations of *Ae. aegypti*. Except for the vaccine against yellow fever, there are no vaccines or drugs to reduce disease burden. This has resulted in the development of novel approaches, including population replacement or population suppression, to control *Ae. aegypti* and associated arboviral diseases [3,4].

Population suppression strategies are based on mass production, sterilization and release of sterile males into the target area to induce sterility in the wild females [5]. Early mosquito population suppression attempts against *Ae. aegypti* were conducted in the 1960s to the 1980s [6] using various sterilization strategies for the released males such as chemo-sterilization, ionizing radiation and chromosomal aberrations [5]. The first field trial against *Ae. aegypti* using gamma-irradiated males failed due to reduced male competitiveness [7]. Subsequent releases of both chemo-sterilized males and *Ae. aegypti* males carrying chromosomal translocations led to various levels of population reduction in the targeted areas [8,9]. Various technical problems such as the low competitiveness and fitness performance of the released laboratory males in the field, immigration of fertilized females into the release areas, inefficiency of the available sex separation systems and lack of institutional commitment to carry on the population control process eventually led to the cessation of population suppression strategies for years [5,6].

The growing concern on *Aedes*-transmitted diseases in the last decade and recent advances in biotechnology have revived the interest in mosquito control. Some of the emerging population suppression strategies based on CRISPR technologies are still at the early stages of development or validation [10,11]. On the other hand, various open-field pilot trials have been recently conducted against *Ae. aegypti* [12–16], *Ae. albopictus* [17–19] or *Ae. polynesiensis* [20], using male sterilization methodologies such as the sterile insect technique (SIT), the incompatible insect technique (IIT), the combined SIT/IIT and the release of insects carrying a dominant lethal (RIDL). *Ae. aegypti* RIDL field trials were performed using the same engineered strain first in the Cayman Islands leading to an 80% population reduction [21], and then in an open-field trial in Brazil where similar mosquito population reduction was achieved [13]. Another study in Thailand used irradiated *Wolbachia*-infected males (combined SIT/IIT) in an open-field trial leading to 84% reduction [15,16], while the recent release of *Wolbachia*-infected males (IIT) at large-scale trials in California achieved up to a 95% reduction of the target population [14].

Irrespective of the male sterilization methodology, mosquito population suppression requires the development of efficient sex separation systems to limit the risk of releasing fertile and/or potentially pathogen transmitting females [3,22]. Several mosquito sex separation methods have been developed over the years based on (i) pupal size or developmental sexual dimorphism—using the smaller size and/or faster development of male pupae to discriminate them from females [14,23]; (ii) adult sexual morphological dimorphism—using male-specific genitalia and antennal features to separate them from females [14]; (iii) behavioural differences such as blood-feeding, where insecticides are added to the blood meals leading to female elimination [24,25]; and (iv) the development of genetic sexing strains (GSS) by linking a selectable trait to the male-determining factor [22,26–29].

Sex separation in *Ae. aegypti* pilot field trials is mostly based on the pupal sexual dimorphism using a mechanical device known as a glass separator [23]. Recently, an automated process was developed by Grupo Tragsa suggesting that pupal size-frequency distribution can be modelled for each given strain/population using an automated pupae size estimator system, that collects pupae size measurements based on artificial vision; this distribution model can be used to improve both purity and performance of current sex separation systems based on actual biometric data of each specific strain [30]. A more recent development uses an automated mechanical sieve that separates male from female pupae based on their size and the emerged adults are further scanned and separated according to sex-specific morphological traits in an automatic camera-based way [14].

The potential damage of mosquitoes and errors during pupal and/or adult handling, environmental concerns and poor male quality due to insecticide use in blood meals, and the increased cost of rearing females until pupal or adult stage in the currently used systems, lead to the conclusion that efficient and robust sex separation methods, ideally in the form of GSS, can be beneficial for sex separation of *Ae. aegypti* [22,29].

GSSs developed through classical genetics require two primary components: a visible or conditional lethal mutation as a selectable marker and its linkage (wild-type allele) to the male-determining locus [31]. In the resulting strain, the males are heterozygous with a normal ‘wild-type' phenotype, while the females are homozygous for the recessive allele of the selectable marker, expressing the mutant phenotype [31]. The most successful example of such a GSS is the VIENNA GSS of *Ceratitis capitata* (medfly) [31,32]. In *Aedes* mosquitoes, male development depends on a dominant male-determining locus (M locus) that resides on the homomorphic sex-determining chromosome 1 (M chromosome) [33–35], with males being heterogametic (Mm) and females being homogametic (mm) [36]. There are many markers that could be used for *Ae. aegypti* GSS development related to eye and body colour and insecticide resistance [37]. Among them, promising markers are the Red-eye (*re*) and the White-eye (*w*) markers, which have been reported to be stable and evidenced early in development, with full penetrance and expressivity, and already sex linked (located in chromosome I) [37–39].

In contrast with medfly (and many Diptera in general), genetic recombination in *Aedes* males poses an additional threat to the genetic stability of such strains, making the incorporation of additional recombination-suppressing factors, such as inversions, essential [31,32]. Different studies in the past have shown that the induction of inversions to suppress recombination in *Ae. aegypti* is a feasible strategy [40]. Research performed recently by our group demonstrated once more the potential of irradiation to induce inversions and provided promising tools to suppress recombination in *Ae. aegypti* [41]. Currently, the model species for GSS development that has supported the worldwide successful application of SIT is the medfly. Its success has been built upon decades of research in entomology and classical genetics that delivered both the last generation of the VIENNA 7 and VIENNA 8 GSS but also accompanying improvements, such as the reduction of recombination through inversions and the development of a filter rearing system (FRS) that guarantee both the preservation of the genetic sexing system integrity and the almost zero contamination of the release batch with females [31,42]. The FRS in particular has been adapted by the major rearing facilities and relies on the principles of (i) keeping a small colony under relaxed conditions and continuous removal of recombinants, if present, to preserve quality and genetic integrity (this is the filtered colony); (ii) use of material from the filtered colony for upscaling through amplification cycles to produce the male release batches; and (iii) never transferring material from the amplification cycles back to the filtered colony [31,42].

Although modern technologies including transposon- and CRISP/Cas9-based approaches are promising to deliver GSS through providing the tools for both targeted mutagenesis and genetic linkage of desirable genes with the M locus [22,28], they are still developing and issues related to regulatory restrictions, public acceptance and sustainability over time and under mass-rearing conditions (long-term monitoring) have to be addressed [43,44]. Therefore, using classical genetic strategies for developing GSS for *Ae. aegypti*, although it may seem ‘old school', offers the benefits of having proven effective for long-term use and has gained public support and regulatory acceptance worldwide.

In this respect, and following the medfly paradigm, here we present the construction of two strains for *Ae. aegypti* that are based on the *re* and the *w* morphological markers and their evaluation under laboratory-scale rearing. At the same time, we present the incorporation of a previously developed inversion (Inv35) and discuss its effects on the genetic stability and fitness of the strains. We also propose a combined filtering approach that is based on pupal size and pupal eye colour and minimizes recombinants that constitute a threat to both the genetic integrity of the strain and female contamination of male release batches. Finally, we present, as a proof of concept, a camera-based image analysis algorithm that can efficiently discriminate pupae sex based on the eye colour and can be used to develop an automated sex-sorting system combined with other already available automated approaches that focus on pupal size.

## 2. Methods

### (a) Mosquito strains

**Figure 1.**
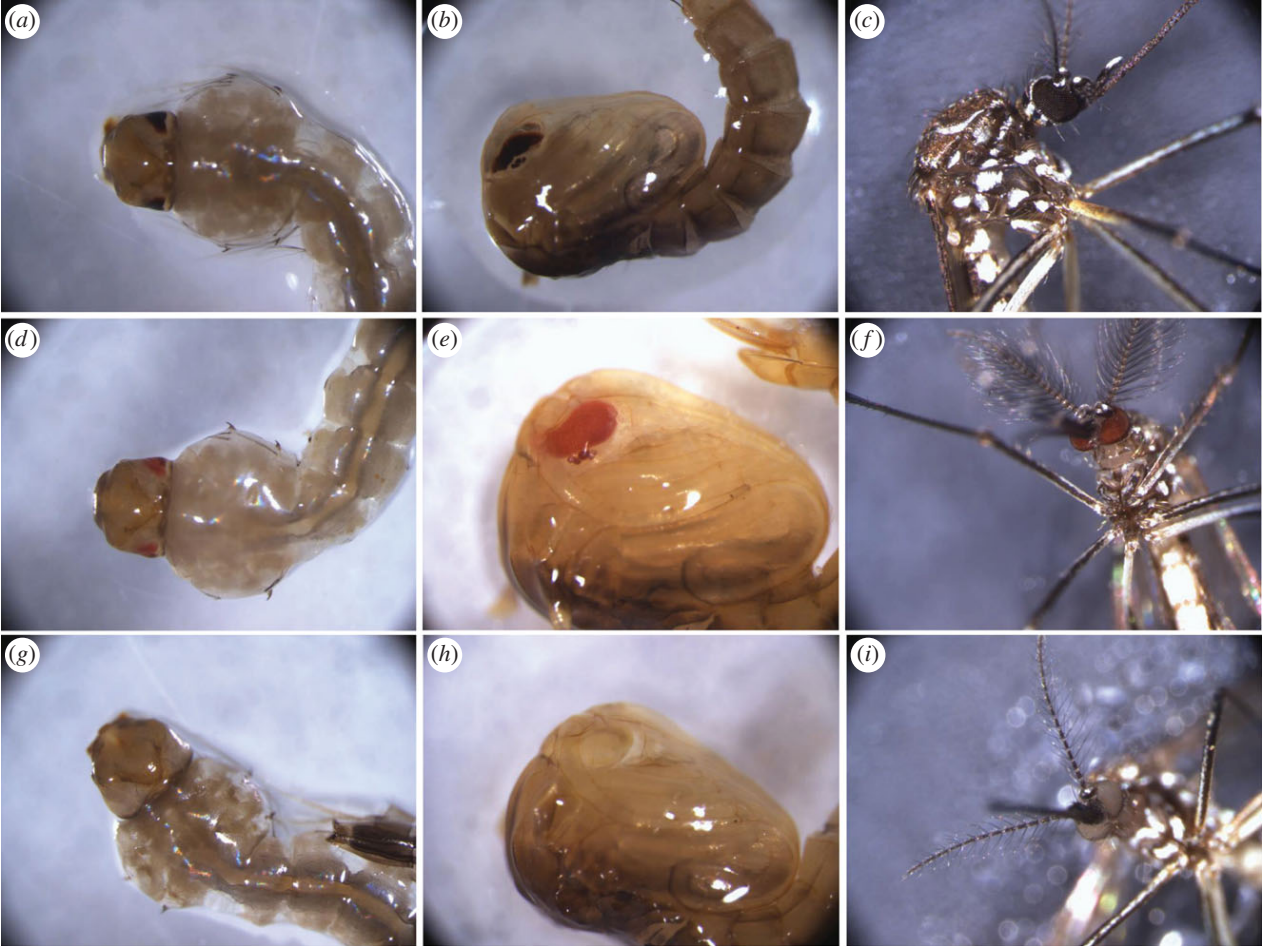
The eye colour of wild-type, Red-eyes and Higgs is dark brown (*a–c*), red (*d–f*) and white (*g–i*), respectively, consistent in larva L3 (*a,d,g*), pupa (*b,e,h*), and adult (*c,f,i*).

Documenting the origin and history of *Ae. aegypti* laboratory strains is not always easy and this may be reflected in strains' properties as well [45]. The Red-eye (*re*), Higgs White-eye (*w*) and a Brazilian wild-type strain (BRA) were used in the present study. The *re* and *w* strains are laboratory strains with a rather well-documented history and have already been used in different studies as markers for genetic transformation, genetic linkage or to test the competence of laboratory strains to Zika virus [46–48]. The ‘BRA' strain is a recently domesticated laboratory population derived from wild populations in Brazil. In the present study, it was used both as the ‘donor’ of the wild-type alleles and to compare the quality of the resulting strains since this laboratory population was established also as a candidate for SIT or combined SIT/IIT releases in the area [49]. The ‘wild-type' (*wt*) colour of the compound and the simple eye in *Ae. aegypti* is dark brown/black and stable during all developmental stages (figure 1*a–c*). The colour of the *re* compound and simple eye is constantly red during all developmental stages (figure 1*d–f*). In newly emerged adults, the eye is a deep blood-red colour (figure 1*f*) that gradually darkens with age. Similarly, the *w* compound and simple eye are white (figure 1*g–i*), and the colour is also stable in all developmental stages. *Ae. aegypti* strains were maintained in the insectary of the Insect Pest Control Laboratory (Joint FAO/IAEA Division, Seibersdorf, Austria) at 27 ± 1°C, 80% relative humidity and a photoperiod of 12/12 h day/night. Adult mosquitoes were kept in standard (30 × 30 × 30 cm) insect-rearing plastic cages (BugDorm-41515 insect cage) with constant access to a 10% sucrose solution. Blood feeding of adult female mosquitoes was performed using porcine blood three times per week. Moistened oviposition papers (white Creped Filter Papers) were inserted into the cages 48 h after the last blood-feeding round to collect the mosquito eggs.

### (b) Inheritance pattern

The inheritance pattern of the *re* and *w* mutations has been extensively studied before [36–39]. However, variations in recombination frequencies have been reported, attributed to a variety of factors including the genomic background of the strains [41,50]. Therefore, inheritance was studied through individual crosses between mutant females and wild-type males and the reciprocal cross between mutant males and wild-type females. The phenotype of F1 offspring was screened for each individual cross separately. Subsequently, F1 males were crossed with F1 females (F1 inbreeding) and F1 male progeny were backcrossed with the parental mutant lines. F2 progeny were screened regarding both the phenotype and the male/female ratio to define the inheritance pattern of the mutation. Families with less than 25 surviving progeny were discarded. For each type of cross, 15 single pairs were set up using newly emerged virgin males and females. Individual crosses were performed by inserting one male and one female in a plastic cup (120 ml). The cups were then covered with a piece of mesh fabric held by rubber bands. Sugar solution (10%) was offered in pieces of moistened cotton on the mesh fabric. Individual oviposition was performed as described previously [51], using 24-well cell culture plates placing one female per well. Subsequently, the eggs were air dried for 48 h and 5 days later transferred to plastic cups (120 ml) that contained a hatching solution at 27 ± 1°C as described previously [52]. The hatched larvae were transferred to small plastic trays (500 ml) that contained larvae diet as reported previously [53]. The progeny of each family were kept separately. Larvae were screened under a stereomicroscope to define the colour of their eyes. Eye colour was also screened in the pupal and adult stage under a stereomicroscope to confirm the consistency of the phenotype. The gender of the offspring was screened in the pupal and the adult stage. Additionally, mass crosses were performed for the two test backcrosses and the offspring were also screened in order to define the inheritance pattern and compare the results with those of individual crosses. *χ*^2^ statistics were used to compare the observed ratio of eye colour phenotypes (wild-type : mutant) in F1 inbreeding crosses against the expected 3 : 1 ratio for a recessive mutation, and the observed sex ratio of mutant progeny in backcrossings against the expected 1 : 1 ratio for a mutation that is not linked to the sex.

### (c) Development of the first generation of genetic sexing strains

#### (i) Red-eye GSS and White-eye GSS

The offspring of the backcross was used to generate a GSS for each mutation. Heterozygous F1 males *(+ M/re m* or *+ M/w m*) were backcrossed with mutant females (*re m/re m* or *we m/we m*) for the development of the Red-eye GSS or the White-eye GSS, respectively. These crosses created strains primarily consisting of parental genotypes (heterozygous wild-type males and homozygous mutant females, red- or white-eye) with a lesser number of recombinant genotypes (mutant males and wild-type females). Two types of colonies were created for each mutation. A filter colony was developed by keeping only the expected progeny of the backcross (*re* or *w* females and wild-type males) and removing the recombinant progeny (wild-type females and *re* or *w* males) (electronic supplementary material, figure S1). This procedure was followed for each new generation using the FRS described below. Non-filtered colonies were maintained for both GSS and the percentage of recombinant progeny was followed and recorded for a series of generations to define the time required for each non-filtered strain to collapse. These colonies were kept by inbreeding, without removing recombinants.

#### (ii) Red-eye GSS/Inv35 and White-eye GSS/Inv35

In a recent effort, chromosomal rearrangements were induced through irradiation [41], and isolation of recombination suppressors (inversions) for the M chromosome was done following the approach previously described [40]. The most promising of them (Inv35) was introduced in the Red-eye GSS and White-eye GSS by crossing wild-type males having the recombination suppressor (from the Inv35 line) with females from the two GSS. Both filtered and non-filtered colonies were developed and maintained for numerous generations, similar to the strains without the inversion.

### (d) Filter rearing system

A FRS was developed for the removal of recombinant progeny that allowed the maintenance of pure Red-eye GSS and White-eye GSS colonies (both with and without the inversion). Sorting was done at four levels: (i) during pupal stage using a glass sorting apparatus [23], exploiting the pupal size sex dimorphism; (ii) during the pupal stage, under a stereomicroscope, after size selection, exploiting the eye colour markers; (iii) during the pupal stage, after pupal size and eye colour selection, by screening the genital lobe (under a stereomicroscope); and (iv) at the adult stage, based on the male antennae that are branched and feathery (visual inspection of emerging mosquitoes). The combined use of different sex-specific or sex-linked characters at different stages augments the effectiveness of the system (electronic supplementary material, figure S1).

### (e) Quality control analysis of the genetic sexing strains

Adequate rearing efficiency and biological quality of a strain are prerequisites prior to scaling up for pilot trials. The following strains were included in the QC analysis: (i) the wild-type Brazil strain (BRA) as control which was used also as the donor of the wild-type alleles for the construction of the two GSSs, (ii) the Red-eye GSS, (iii) the White-Eye GSS and (iv) the Red-eye GSS/Inv35 to assess the effect of the inversion on the genetic stability and selected QC parameters. Due to inferior quality documented during initial QC experiments, a partial QC was performed for the White-eye GSS/Inv35, mainly to test the effect of the inversion on the genetic stability.

#### (i) Immature development

Three replicates of 400 eggs were counted on wet filter papers after a 48 h collection and subsequently air dried for 48 h. Hatching was done as described above [52]. Ten millilitres of the larval diet were provided daily in each container. All containers were observed once per day (08.00) and pupae were removed and transferred into small plastic trays in cages for emergence.

#### (ii) Recovery rate

Hatching was calculated as the larvae that survived until at least L4. Egg to pupa, egg to adult and pupa to adult recovery were recorded, as well as sex ratio (determined both on pupal and adult stages). Upon the development of the Red-eye GSS/Inv35, a smaller experiment comparing it only with the Red-eye GSS was performed, using (i) three replicates of 100 eggs of each strain to directly assess hatching and (ii) three replicates of 100 L1 per strain to assess downstream productivity.

#### (iii) Immature stages' developmental duration

Pupae were collected once per day (8:00), removed from the tray and kept as different batches. Adult emergence was counted separately for each batch, once per day (8:00).

#### (iv) Pupal weight

This was applied as a quick and easy way to the proxy measurement for adult size and to document the presence of pupal size sex dimorphism. Measurement was performed for pupae 1–24 h old and after drying for 30 min. Five batches of 10 pupae each were measured per sex and strain.

#### (v) Adult longevity

Forty-five to 55 newly emerged adults were put in 15 × 15 × 15 cm cages. Adult lifespan was determined using a 10% sugar solution as food and water source. Dead adults were removed and recorded daily for the first 30 days of the experiment and every 5 days for the next 45 days (total duration of 75 days). Three replicates were performed per sex.

#### (vi) Female fecundity

Twenty males and 20 females were placed in a 15 × 15 × 15 cm cage upon emergence and allowed to mate. After 3–4 days, females were transferred to another cage where they were blood-fed (mass) for two consecutive days (at least two blood feedings of at least 15 min each). Those that were effectively fed (visually inspected) were put in 15 × 15 × 15 cm cages, in replicates of five females. At least three replicates per strain were performed. After 2–3 days, females were provided with egg-laying devices that were left in the cages for at least 60 h. After the first gonotrophic cycle, females were collected in a single cage for blood-feeding, and the same procedure was repeated for the second gonotrophic cycle. Eggs from the two gonotrophic cycles were counted separately. Fecundity was measured as the mean number of eggs laid per female per gonotrophic cycle.

#### (vii) Flight ability

This was performed as previously described [54]. Briefly, batches of 90–110 males, 3–5 days old, were used per test.

### (f) Measurements of genetic stability—recombination screening

#### (i) Red-eye genetic sexing strains and White-eye genetic sexing strains

Recombination frequencies were measured in eleven consecutive generations for the Red-eye GSS (F1–F11) and nine consecutive generations for the White-eye GSS (F1–F9) that were kept under filter conditions. Recombination frequencies were measured for consecutive generations for the respective unfiltered GSSs until the accumulation of recombinant genotypes. To determine parental age effect on recombination frequencies of the two filtered GSSs, recombination was measured in progeny deriving from different gonotrophic cycles separately, when possible (GC1–GC3).

#### (ii) Red-eye genetic sexing strains/Inversion 35 and White-eye genetic sexing strains/Inversion 35

Following the introduction of the Inv35, recombination frequencies were measured for four generations (F1–F4) for the White-eye GSS/Inv35 both under filtered and non-filtered conditions. We stopped continuous recording in F3 since the accumulation of recombinants in the non-filtered colony was already evident. On the other hand, recombination frequencies for the Red-eye GSS were monitored for eleven generations (F1–11) both under filtered and non-filtered conditions since the accumulation of recombinants was not detected.

### (g) Selection of radiation dose and downstream experiments with irradiated males

#### (i) Radiation dose-response curve

At least 99% induced male sterility is needed for the application of SIT [55]. To evaluate the minimum dose required to achieve this level, Red-eye GSS males of age 30 to 36 h post pupation were divided into six groups for the six radiation doses (0, 30, 50, 70, 90 and 110 Gy) using the method described as a stackable Petri dish canister in a Gammacell 220, self-shielded, gamma-ray Cobalt 60 irradiator [56] with a dose rate from 1.470 to 1.443 kGy m^−1^. Dosimetry was performed according to standard operating procedures regarding dosimetry systems for SIT [57] and all readings were within the 95% confidence intervals. The irradiated male pupae emerged in BugDorm-1 cages (30 × 30 × 30 cm), with access to 10% sucrose solution. After adult emergence, each cage received 60 virgin adult females and 60 irradiated virgin males. The adults mated for 2 days and females received a blood meal for at least three consecutive days. Moistened filter paper was placed inside each cage and left for 2 days for egg collection. Egg cups were removed from cages dried for 3 days, counted under a stereomicroscope and immediately placed to hatch. The hatch rate of each dose was determined as the total number of larvae divided by the total number of eggs.

#### (ii) Flight ability of irradiated males

To test how irradiation affects male flight ability, male pupae irradiated with 90 Gy (30–36 h after pupation) were left to emerge and a flight ability test was performed using 3–5 days old males as described previously [54].

#### (iii) Male mating competitiveness

Red-eye GSS males 30 to 36 h post pupation were placed in the irradiation canister and irradiated at 90 Gy (as described above). After irradiation, male pupae were placed in BugDorm-1 cages for adult emergence. Males that emerged from both irradiated Red-eye GSS pupae and wild-type (BRA) pupae were placed simultaneously in BugDorm-1 cages while wild-type (BRA) females were added at least 30 min after the males. The ratio of males and females in these cages was 1 : 1 : 1 and 10 : 1 : 1 (♂irradiated GSS:♂wt:♀wt) with a total number of 250 insects per cage to control stress due to density, including the control crosses at a ratio of 1 : 1 (♀:♂)—one control using only fertile wild-type males (CTRL-WT) and the second only sterile Red-eye GSS males (CTRL-ST). Both groups of males mated with wild-type (BRA) females. Adult feeding, blood-feeding, egg collection and hatching was performed as described above. The number of eggs was recorded and the hatch rate was determined. The male competitiveness index (*c*) was calculated using the formula c=N/S∗(Hn−Ho)/(Ho−Hs), where *N* is the total number of wild-type males, *S* is the total number of sterile males, *Hn* is the wild-type hatch rate (CTRL-WT cage), *Hs* is the sterile hatch rate (CTRL-ST cage) and the *Ho* is the observed hatch rate of each ratio. The residual fertility was obtained as the percentage of the hatch rate from each ratio in relation to the control cage and was subtracted from 100% to calculate the induced sterility index (ISI) [58].

#### (iv) Cage population suppression

Similar to the male mating competitiveness experiment, treatment and control cages were set up to simulate a population suppression event [59]. We followed the principles presented for testing the ability of transgenic (RIDL) *Ae. aegypti* and medfly strains to suppress cage populations [60,61], incorporating additional modifications described for the cage suppression experiments in *Ae. albopictus* using the combined IIT/SIT approach [19]. A few additional modifications are specified below. Either Red-eye GSS or Red-eye GSS/Inv35 adult males (irradiated as described above) were released in equal numbers twice a week, with the second release taking place 48 h after the first one. Unlike Zeng *et al.* [19], cages were set up with a maximum of 250 mosquitoes (in standard 30 × 30 × 30 cm BugDorm-41515 insect cages) to avoid overcrowding. The released sterile males were 3 to 4 days old at the first release and 5 to 6 days old at the second release. Three types of cages (with three replicates each) were initially setup. The following ratios were used: (i) the 1 : 1 : 0 cages, which represents the ‘fertile control cage', consisting of 1 wild-type female to 1 wild-type male to 0 irradiated Red-eye GSS males; (ii) the ‘1 : 1 : 1' cages; and (iii) the ‘1 : 1 : 10' cages. The number of fertile adults in the suppression cages (1 : 1 : 1' and ‘1 : 1 : 10) was adjusted by multiplying the observed hatch rates in the control cages by the respective ones in the suppression cages and this would be the percentage of pupae (fertile male and female) composing the next round. Slightly modified from Zeng *et al.* [19], to reduce the rearing time until next generation, the rearing was anticipated, so by the time the adjusted hatch rate was determined, the adults would be ready to restock the respective cages, and in addition, larvae were returned in the control cages based on their hatching rate and not at a standard rate. Egg laying and hatching as well as larva and adult handling and feeding were performed as described above.

### (h) Identification of red eyes through image analysis

The identification of red eyes is based on an image acquisition and analysis system which takes advantage of the fact that melanized black eyes in the males appear as a black BLOB (Binary Large Object) under near-infrared light (850 nm), while red eyes cannot be seen due to the lack of melanin. The algorithm performs a morphological analysis in several steps, such as segmentation of body, identification of centroid and alignment of the cephalothorax and search of dark BLOBs in the area defined by the relative positions of the latter. The pictures of the pupae used for this analysis have been taken using a camera model ‘IDS UI-3080CP-C-GL Rev 2' with no IR filter, a ‘RICHO FL-CC2514-5M' lens and a source of IR light with a wavelength of 850 nm. The application that implements the algorithm has been developed in C++ language, using OpenCV 3.4.2 graphics library and Visual C++ 2017. As a proof-of-concept, the developed algorithm was applied on two different batches: (i) 50 randomly selected pupae and (ii) a random sample of 512 pupae (256 male and 256 female) including 24–48 h old pupae. Regarding computational power, we used an Intel(R) Core(TM) i7-7820HQ CPU @ 2.90 GHz, 2801 Mhz processor with a 16 Gb RAM. Detailed information and full algorithm in C++ are presented in the electronic supplementary material figures S5 and S3.

### (i) Statistical analysis

The analysis of hatch rate, downstream productivity (egg to L4, egg to pupa, egg to adult, pupa to adult, L1 to pupa and L1 to adult recovery), sex ratio, male flight ability and average number of eggs per female was performed with one-way ANOVA. All assumptions to use ANOVA were met (independence of observations, normal distribution of dependent variable and homogeneity of variances). The effect of the gonotrophic cycle and the strain on the average number of eggs per female was analysed with two-way ANOVA. The effect of the irradiation, strain and their combination, on male flight ability, was tested with two-way ANOVA. For the analysis of duration of immature stages development and adult lifespan, the Kaplan–Meier method was used. All statistical analyses described above were performed at the significance level of 0.05 using SPSS v.24. Chi-square P-values were calculated using Microsoft Excel 2016 formulae. For experiments including irradiation dose–response curve, irradiated male mating competitiveness and cage suppression, statistical analysis was conducted in RStudio using R background with the following packages: tidyverse, rcompanion, EnvStats and ggpubr. For differences between groups, a generalized linear model was used. All the statistical analysis done with R is presented in the electronic supplementary material, S2.

## 3. Results and discussion

### (a) *Re* and *w* markers: inheritance pattern

The *re* and *w* mutations have already been mapped to chromosome I [39], making them suitable markers since the induction of chromosome I-autosome translocations is not needed. The full penetrance and expressivity of both mutations, along with their stability during development was verified (figure 1). In this study, the eye colour was evident through development, starting from L1 up to the adult stage. An exception was observed in old pupae (close to emergence) where both the Red-eye and the White-eye darkened thus making separation from the wt eye difficult (although still possible).

### (b) Recombination frequencies and development of the Red-eye and White-eye genetic sexing strains

**Figure 2.**
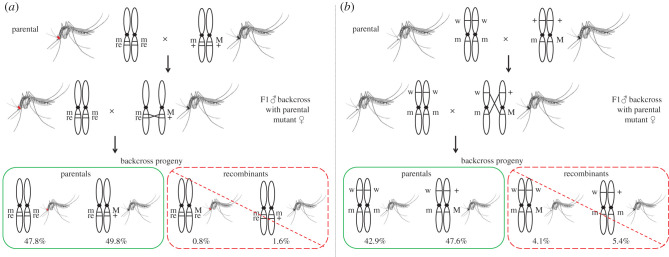
Backcrossing scheme for the assessment of recombination rates between *re* or *w* and M locus and the construction of Red-eye GSS and White-eye GSS. (*a*) Wild-type BRA males *+ M/+ m* were crossed to virgin Red-eye females *re m/re m.* F1 males *+ M/re m* were backcrossed to virgin Red-eye females and backcross progeny were screened for sex and eye colour. Red-eye females and wild-type males are the parental genotypes (circled with green), while Red-eye males and wild-type females are the recombinant genotypes (circled with red). (*b*) Wild-type males *+ M/+ m* were crossed to virgin Higgs females *w m/w m.* F1 males *+ M/w m* were backcrossed to virgin Higgs females and backcross progeny were screened for sex and eye colour. White-eye females and wild-type males are the parental genotypes (circled with green), while white-eye males and wild-type females are the recombinant genotypes (circled with red). (Online version in colour.)

The genetic distance of both mutations with the M locus was measured through reciprocal genetic crosses (figure 2). All individual bidirectional crosses (38 for the *re* and 30 for the *w*) showed that the *wt* alleles were always dominant over *re* and *w* alleles (electronic supplementary material, table S1). Inbreeding of individual F1 families (18 involving the *re* and 27 involving the *w* marker) showed the expected phenotypic ratios in F2 (electronic supplementary material, table S1). Recombination was measured between the *re* and the M locus in two bidirectional en masse crosses and 21 families derived from the bidirectional single pair crosses (nine and 12 from each direction) and was found to be 2.4%, 1.3%, 1.9% (±0.82) and 2.3% (±0.61). Similarly, recombination was measured between the *w* and the M locus in two bidirectional ­en masse crosses and 24 families (11 from each direction) and was found to be 9.6%, 13.0%, 11.35% (±1.7) and 14.77% (±1.3) (electronic supplementary material, table S2). Therefore, we confirmed that (i) the inheritance of both mutant phenotypes is controlled by sex-linked, recessive genes as shown in previous studies [37–39]; (ii) recombination frequencies of both mutations (*re* and *w*) in respect to the M locus are in the range of those previously described [36,38,39,41,50]; (iii) there is variability in recombination rate which can be attributed to different factors, such as random variation and small genomic differences; and (iv) the recombination frequency in males and females is comparable.

Based on that, we developed two different strains, named Red-eye GSS and White-eye GSS, using the genetic crosses shown in figure 2. The first backcrossing of the Red-eye GSS generated the expected *re* females and *wt* males (47.8% and 49.8%, respectively, figure 2*a*) and the recombinant *re* males and *wt* females (0.8% and 1.6%, respectively, figure 2*a*). Higher recombination rates were obtained for the White-eye GSS with a total of 9.5% recombinant progeny (figure 2*b*). Recombination frequencies were in the range of those previously described for these mutations and the M locus [36,37,41].

### (c) Development of a filter rearing system

A FRS was developed (electronic supplementary material, figure S1) allowing the maintenance of a filtered colony for each GSS by inbreeding after removing the recombinant progeny. For the filtered Red-eye GSS, 27 495 mosquitos were screened for 11 consecutive generations (electronic supplementary material, table S2). Occasionally, we identified either eye colour recombinants (electronic supplementary material table S2) or pupae that were not properly sorted with the glass sorter (in the expected range of 1/1000–1/10000). However, after the application of both pupal size glass sorting and pupal eye colour visual inspection, zero mosquitos were assigned to the wrong sex, as demonstrated by the subsequent screening based on the pupal genital lobes and adult plumose antennae (electronic supplementary material figure S1). Therefore, the combined use of the two filters successfully eliminated both eye colour recombinants and pupae that were not correctly separated during glass sorting, making a visual inspection of the additional characters rather redundant. That was not the case for the White-eye GSS, since there were sporadic escapers from both filters in the 9772 mosquitos screened from nine consecutive generations (electronic supplementary material, table S2). This is attributed to the low efficiency of the pupal eye colour sorting as a result of the high recombination frequency (electronic supplementary material, table S2).

### (d) Factors affecting recombination frequencies

The recombination frequencies were stable across generations for both the Red-eye GSS filtered colony, ranging between 1–2.5% and the White-eye GSS filtered colony, ranging between 9–13% (electronic supplementary material, table S2). Non-filtered colonies were also maintained for both strains. After eight generations in the non-filtered Red-eye GSS colony, recombinant genotypes gradually accumulated to greater than 10%. No male or female bias of recombinants was evident (the *re* and *wt* phenotypes were in equilibrium and approximately 50% each), indicating that red-eye mutation is probably not associated with a significant fitness cost. On the other hand, in the non-filtered White-eye GSS colony, recombinant genotypes accumulated faster, leading to the collapse of the strain within four generations. The increased accumulation of female recombinants (in contrast with males), indicates that the specific mutation is associated with a fitness cost, since the percentage of *w* phenotypes gradually decreases in the colony.

Different factors have been reported as potentially affecting recombination frequencies, including the genomic background, age of the mosquitos and fluctuations in the environmental conditions [50]. However, the effect of these factors has not been systematically tested. In our case, a random fluctuation of recombination among generations was observed*,* with fluctuation being in the expected range (electronic supplementary material, table S2) [37,50]. Data from different gonotrophic cycles of the same generation did not point to a parental age effect on male recombination (electronic supplementary material, table S2).

### (e) Quality control measurements of the Red-eye and White-eye genetic sexing strains

Although the ultimate test is the field performance against target populations and laboratory experiments may overestimate the potential of strains to be used for SIT purposes, it is important to characterize strains prior to upscaling to detect any constraints related to rearing efficiency and biological quality. The actual testing of the competence of a strain can derive only from semi-field experiments against populations recently collected from the wild or small-scale SIT trials against the target populations. To assess the fitness of the strains, which is important for the mass production of the insects to be released in the field, several parameters were tested for both GSSs. Our strains were constructed by crossing the old laboratory strains carrying the *re* and *w* mutations with the wild-type ‘BRA' strain collected recently from Brazil and considered as a candidate strain for SIT applications in the area. Therefore, the biological quality of the two GSS was tested against the ‘BRA' strain. Comparison with the ‘BRA' strain was used to test for basic quality control characters that can provide evidence in favour or against of upscaling a strain to be tested for SIT purposes. Quality control for both Red-eye and White-eye GSS is summarized in electronic supplementary material, table S3. There were no significant differences among the three strains in respect to sex ratio and immature development duration of both sexes. However: (i) the White-eye GSS demonstrated significantly reduced productivity compared to both BRA and Red-eye GSS (egg to adult recovery: *F* = 9.361, d.f. = 3 and *p* < 0.05) and (ii) Red-eye GSS showed significantly higher lifespan (males: Chi-Square = 22.801, d.f. = 3 and *p* < 0.05; females: Chi-Square = 159.413, d.f. = 3 and *p* < 0.05) and significantly better flight ability (*F* = 61.059, d.f. = 2 and *p* < 0.05) than the BRA strain.

### (f) Biological quality of irradiated males: flight ability, mating competitiveness and cage population suppression experiments

**Figure 3.**
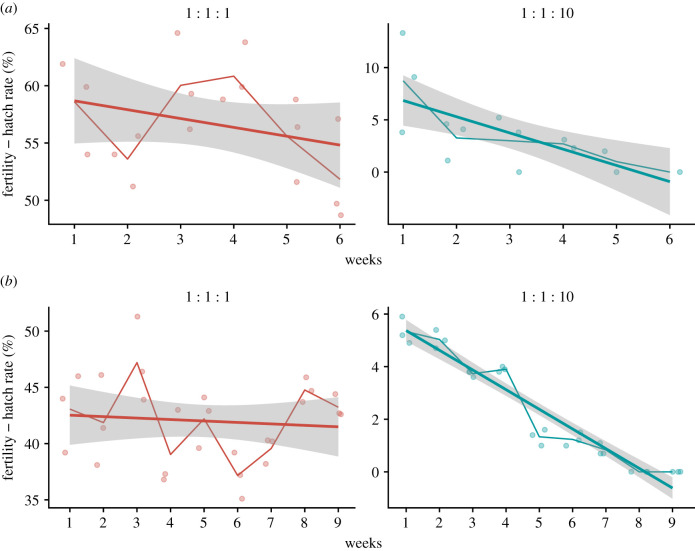
The cage suppression experiment. The Red-eye GSS (*a*) and the Red-eye GSS/Inv35 (*b*) were tested for their ability to suppress a target population in laboratory cage experiments by setting up three types of cages with three replicates each: the ‘1 : 1 : 0' fertile control cage (1 wild-type female: 1 wild-type male: 0 irradiated Red-eye GSS males), the ‘1 : 1 : 1' suppression cage and the ‘1 : 1 : 10' suppression cage. Only the two later are presented (‘1 : 1 : 1' and ‘1 : 1 : 10’) since the data from the fertile control cage have been used to assess the baseline fertility used for the calculations. Releases of irradiated Red-eye GSS were performed twice a week for six weeks, and releases of Red-eye GSS/Inv35 males were performed twice a week for a period of nine weeks. The thicker straight line represents the generalized model with the grey-shaded area as the standard error, while the jagged thinner line represents the mean of each week; the points indicate the observed data. (Online version in colour.)

The application of SIT also depends on the ability of the released irradiated sterile males to compete for mating with wild-type females against wild-type males. The optimal dose for induced sterility was determined by testing different radiation doses and developing a radiation dose–response curve (electronic supplementary material figure S2), which led to the selection of 90 Gy for downstream experiments (adequate to induce sterility of at least 99% in the Red-eye GSS in accordance with WHO and IAEA recent guidelines) [55]. Flight ability and mating competitiveness of irradiated males (electronic supplementary material, table S3 and figure S3) demonstrated that the Red-eye GSS was (i) better than the White-eye GSS and BRA regarding flight ability (*F* = 56.488, d.f. = 1, *p* < 0.05) and, (ii) similar to BRA regarding mating competitiveness. More specifically, the ‘1 : 1 : 1' and ‘1 : 1 : 10' ratios had 55.5% and 15% fertility, respectively (GLM *F* = 661.8, d.f. = 3, 20, *p* < 0.05). The calculated Fried Index (mating competitiveness) had a geometric average of 0.53 (s.e. = 0.08) and 0.44 (s.e. = 0.04) for the ‘1 : 1 : 1' and ‘1 : 1 : 10' ratio, respectively, with an Induced Sterility Index (ISI) based on the geometric mean of 34.3% (s.e. = 3.19) and 81.7% (s.e. = 1.34), respectively, suggesting that a release ratio of ‘1 : 1 : 10', which is a realistic scenario for SIT, can be tested to suppress target populations. Therefore, the Red-eye GSS was tested for its ability to suppress a target population in laboratory cage experiments. Compared to the ‘1 : 1: 0' fertile control and ‘1 : 1 : 1' suppression cages, there was a statistically significant reduction in the egg hatch rate in the ‘1 : 1 : 10' cage over time which ultimately resulted in the collapse of the targeted population within six weeks (*F* = 769.2, d.f. = 2, *p* < 0.05) (figure 3*a*, electronic supplementary material S4). Therefore, irradiated Red-eye GSS males can be considered for SIT applications to suppress or even locally eliminate *Ae. aegypti* populations. The mean induced sterility estimated from the ‘1 : 1 : 1' cages was 0.35 giving a mating competitiveness of 0.35 across releases (electronic supplementary material S4). Despite this induced sterility, we did not observe a reduction in the cage population during the six weeks of releases in the ‘1 : 1 : 1' cages. It is possible that more time is needed using larger sample sizes to demonstrate the effect of this ratio. However, this was beyond the scope of this project since such ratios (1 : 1 : 1) are not a ‘realistic' SIT scenario where overwhelming numbers of sterile mosquitos are used (closer to ‘1 : 1 : 10' and even higher).

### (g) Enhancement of genetic stability of the Red-eye genetic sexing strains and the White-eye genetic sexing strain using Inversion 35

Genetic recombination constitutes a concern for both the genetic stability of a GSS and the female contamination of male-only releases. In a recent study of our group, we tried to isolate irradiation-induced recombination suppressors on the M chromosome [41] following the approach described by Bhalla [40]. A promising recombination-suppressing inversion (Inv35) was isolated. In the present study, this inversion was incorporated in the Red-eye GSS and the White-eye GSS, giving the Red-eye GSS/Inv35 and the White-eye GSS/Inv35 strains, respectively. Although the White-eye GSS is not considered a candidate strain for upscaling, due to rearing plus biological quality deficiencies identified both in previous studies and during the QC performed in this study, it was combined with the Inv35 line to further elucidate the effect of Inv35 on the recombination frequency of the genomic region harbouring the *re-*M-*w* loci. Although not eliminating recombination, Inv35 was found to consistently reduce it in both strains (electronic supplementary material, table S2). As evident from the screening of numerous Red-eye GSS/Inv35 mosquitoes (*N* = 21472) for 12 consecutive generations, this strain demonstrated reduced recombination frequencies compared to the original Red-eye GSS (ranging between zero and 0.46% per generation, with an average of 0.22%) (electronic supplementary material table S2 and figure S4). Results were even more encouraging when the strain was under non-filtered conditions, since there was no severe accumulation of recombinants even after 12 generations (ranging between zero and 0.68%, with an average of 0.43%) (electronic supplementary material table S2, figure S4) (electronic supplementary material, table S2, figure S4). Analysis of the data derived from the White-eye GSS/Inv35 are consistent with the presence of an inversion that covers a large part of the region spanning the *re -* M - *w* loci. The average recombination during four generations was 1.93% (range 1–2.78%, *N* = 10 710), which is significantly decreased when compared to the 9–13% of the original White-eye GSS (electronic supplementary material, table S2 and figure S4). Interestingly, the incorporation of the Inv35 in this strain delayed the accumulation of recombinants without filtering as well, since they were 5.61% after four generations; compared to that, the original strain had already collapsed at the same point (electronic supplementary material, table S2 and figure S4).

### (h) Biological quality of the Red-eye genetic sexing strains/Inversion 35

Quality control analysis was performed for the Red-eye GSS/Inv35 and compared to the original Red-eye GSS (electronic supplementary material, table S3). Our data show that the inversion had a cost and reduced (i) productivity (reduced by approximately 16%; *F* = 9.361, d.f. = 3 and *p* < 0.05) and (ii) male flight ability, both with and without irradiation (*F* = 26.126, d.f. = 1, *p* < 0.05 and *F* = 51.187, d.f. = 1, *p* < 0.05, respectively). On a positive view, the flight ability of the Red-eye GSS/Inv35 was comparable to the BRA strain (electronic supplementary material, table S3). To further assess the potential of this strain, its ability to suppress a target cage population was measured similarly to the original Red-eye GSS. A significant reduction in egg hatch was shown in the 1 : 1 : 10 ratio, resulting in the collapse of the target population within nine weeks (figure 3*b*; electronic supplementary material, S4), indicating the potential of this strain to be used in SIT applications. Like above, the mean induced sterility, estimated from the ‘1 : 1 : 1' cages, was 0.41 across releases (electronic supplementary material, S4). Again, we did not observe a reduction in the cage population during the nine weeks of releases with this ratio (figure 3*b*). We observed fluctuations across releases, and it seems that more time is needed using larger sample sizes to demonstrate the effect of this ratio. However, as already stated, the 1 : 1 : 10 ratio is a better simulation of an SIT experiment (with overflowing sterile males).

### (i) Towards an automated, camera-based, sex separation methodology, based on the colour of the eye at the pupal stage

**Figure 4.**
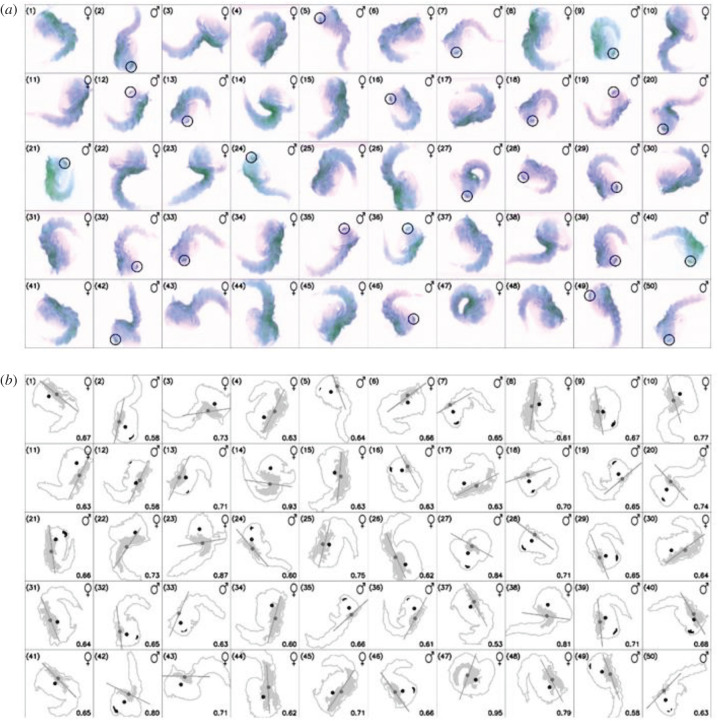
Identification of the eye colour. (*a*) Automatic pupal sex determination is shown in the top right of each box. The position of the eyes in males is also shown. (*b*) Detail of the contour, dorsal parts of the cephalothorax, centroids of the entire contours (black dot) and cephalothorax (dark grey dot), computed orientation lines and BLOBS that have overcome the filtering process are shown, and these features are used to determine the gender of the pupa. The value in the lower right corner of each box represents the circularity index of the convex hull of the dorsal part of the cephalothorax (‘perfect circularity' being 1). (Online version in colour.)

**Figure 5.**
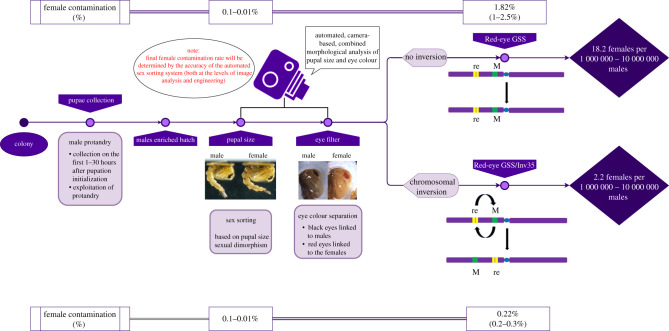
A hypothetical pipeline combining different sex separation strategies to eliminate female contamination in male release batches. The pipeline incorporates male protandry, pupal size dimorphism and pupal eye colour dimorphism. Male protandry is already exploited in mass-rearing facilities and results in batches that are enriched in males in the range of 70–80%. Pupal size sorting performed by skilled technicians using the Fay and Morlan adjustable glass plates system results in contamination rates of 0.1–0.01% with a speed of 15 000–20 000 males per hour. Incorporation of the red eye in the sexing strategy can reduce female contamination by a factor of 1–2.5% (independent markers) while the introduction of Inv35 can reduce this factor to 0.22%. Such a strategy, supported by the appropriate technical advances, can result in female contamination ranging between 2.2 females per million males and 2.2 females per ten million males. (Online version in colour.)

Sex separation in *Ae. aegypti* that is based on pupal size dimorphism is rather time-consuming, labour-intensive and prone to human errors [22]. Moreover, its accuracy can be influenced by varying rearing conditions between larval rearing trays, if not properly standardized. Currently, the productivity of the sex-sorting process done manually by skilled technicians with the Fay and Morlan adjustable glass plates system in a mass-rearing facility ranges between 15 k and 20 k male pupae per hour. We investigated whether the eye colour could be used as a marker to develop a more accurate and potentially automated sexing system. Under a powerful light in the infrared (IR) spectrum, the melanized eye of an *Ae. aegypti* mosquito pupa is seen as a dark binary large object (BLOB) that can be visually distinguished from the rest of the body. Conversely, the red eye of the females of the Red-eye GSS is lacking melanin and therefore is transparent to the IR light. An algorithm was developed and as a proof-of-concept applied on 50 randomly selected pupae (1–24 h old), which allowed the distinguishing of males from females based on a camera-based morphological analysis and the recognition of black eyes in the pupae in a lateral position with 100% efficiency (figure 4 and electronic supplementary material, figure S5). To further assess the efficacy of this strategy and having seen that the pupal eye colour darkens with age, a more extended sample (256 male and 256 female pupae) was analysed, containing 24–48 h old pupae. More than 25 000 images from this batch were analysed. Using the computational power specified in Methods, the average executional time per individual was 5.597 (s.d.: 0.792) msec for the males and 6.699 (s.d.: 1.991) msec for females. In the analysis of red eyes detection, the female contamination was 2.88% (sensitivity > 97%) and the male recovery (specificity) was 80%. It is encouraging that the detection speed is high and that the error was not due to the algorithm itself but to dark pupae that showed abnormal dark spots in the images of the cephalothorax, attributed mainly to female pupae that were close to emergence. We do not expect this to be a real concern under a mass-rearing set-up, since facilities are exploiting the male protandry along with the pupal size dimorphism to reduce female contamination of the release batches. However, a better estimation of the pupae age-dependent darkening on the algorithm's efficiency must be performed prior to full automatization and upscaling. We believe that this sex-sorting method can be acceptable if combined with both a pupae size algorithm and the usual practices of mass-rearing facilities to exploit male protandry (figure 5).

## 4. Concluding remarks

Development of sex-sorting methods that are accurate, robust and amenable to a mass-rearing setup is important to safely and efficiently apply population suppression methods that rely on induced sterility such as, among others, the SIT, the IIT, their combination and RIDL against *Ae. aegypti*. Novel technologies, both in terms of biotechnology, engineering and machine learning, are employed in the race for developing proper sex-sorting strategies. Such methodologies or their combination can provide definite answers to the critical question of population suppression approaches, which is maximizing rearing efficiency and biological quality of released males and eliminating females from the release batches. As for all novel strategies, they need time and effort to address different kinds of concerns before being adopted (including sustainability over time and during mass rearing, public acceptance, regulatory issues and cost).

Development of genetic sexing strains using classical genetics is providing an alternative that bypasses some of these concerns. Strains constructed with similar approaches have been applied universally for medfly control and there is high public acceptance, reduced operational cost, documented efficiency and sustainability, and there are no regulatory issues. Therefore, similar strains developed for *Ae. aegypti* can be easily adopted by mass-rearing facilities, if proven efficient.

Before implementation, a critical question that should be answered is the additive value and the perspective of incorporating a certain element (strain, strategy, equipment) in a mass-rearing pipeline. Here, we present a genetic sexing strategy that is based on the red-eye morphological marker already present on *Ae. aegypti.* This marker is different from those already exploited in sex separation (male protandry, pupal size, genital lobes and adult plumose antennae) and is not affected by mass-rearing conditions, meaning that it can be easily combined with them providing enhanced sex separation accuracy. In our strategy, the incorporation of Inv35 in the Red-eye GSS resulted in an average recombination frequency of 0.22% across 12 generations. Although this may seem high, the fact that there is no accumulation of recombinants for several generations that recombinants were not removed shows that a mass-rearing facility can easily adopt a FRS similar to the one of the medfly to preserve the genetic integrity of the strain and amplification cycles to produce male batches that still have a standard percentage of female contamination. Based on our data, assuming female contamination of 0.22% due to genetic recombination and combined with a standard (commercially available) pupal size sorting method (female contamination ranging between 0.1% and 0.01%) then we get a probability of female contamination ranging between 2.2 females per 1000000 or 10000 000 males, provided that a perfect red-eye sorting system can be developed (figure 5).

Another critical question is how different methodologies can be applied in mass rearing. Towards this direction, we developed a camera-based approach with an algorithm that efficiently differentiates the wild type and the red eye. In terms of computing time to perform the image analysis algorithm, 1 h of work is enough to analyse enough pupae for production above 360 k male pupae (assuming a richness of males in the batch of 75% due to protandry and a male recovery of 80% to reduce the sexing error to the values presented before). The main limitation in terms of productivity is not expected to be the image analysis but the serialization and sorting part of the process. This system can be combined with the algorithms and engineering developments already developed for pupal size discrimination to maximize efficiency. Future steps include testing of this algorithm on larger samples and developing (or choosing) the proper technological equipment to support efficient sorting following the computer-based identification. In any case, the system is scalable, and several unattended sex-sorting units could be used in parallel in a mass-rearing facility if needed.

Sex separation methodologies currently available for *Ae. aegypti* focus on pupal and adult stage dimorphisms. An ideal strategy would eliminate females early during development (eggs or early larvae). The red-eye mutation evidences itself early during development (late L1–early L2), as a clear red spot on the transparent larval body. Although we did not perform any experiments on this, it is worth investing in a strategy that could eliminate most of the female larvae based on the eye colour at early developmental stages. Such an approach would have a huge impact on the logistics of the mass rearing, in terms of diet and larval rearing space plus equipment.

## Supplementary Material

Supplementary Figures S1-S5 and Supplementary Tables S1-S3

## Supplementary Material

Statistical Report for the Irradiation Studies using the Ae. aegypti Red-eyes GSS

## Supplementary Material

Detection of the Red Eye Marker for Sex Identification of Aedes Pupae

## Supplementary Material

Raw data and results from the cage population suppression experiment

## Data accessibility

All data generated during this study, R statistics applied and algorithms developed are provided either in the manuscript or in its electronic supplementary information.
