# Supplementary material for: Genetic sexing strains for the population suppression of the mosquito vector Aedes aegypti
Source: Philos Trans R Soc Lond B Biol Sci. 2020 Dec 28;376(1818):20190808. doi: 10.1098/rstb.2019.0808 (PMC7776939; doi:10.1098/rstb.2019.0808)
Supplement: Supplementary Figures S1-S5 and Supplementary Tables S1-S3 [file rstb20190808supp1.pdf]

Supplementary material

Electronic Supplementary Material 1: Supplementary Figures S1-S5 and Supplementary Tables S1-S3

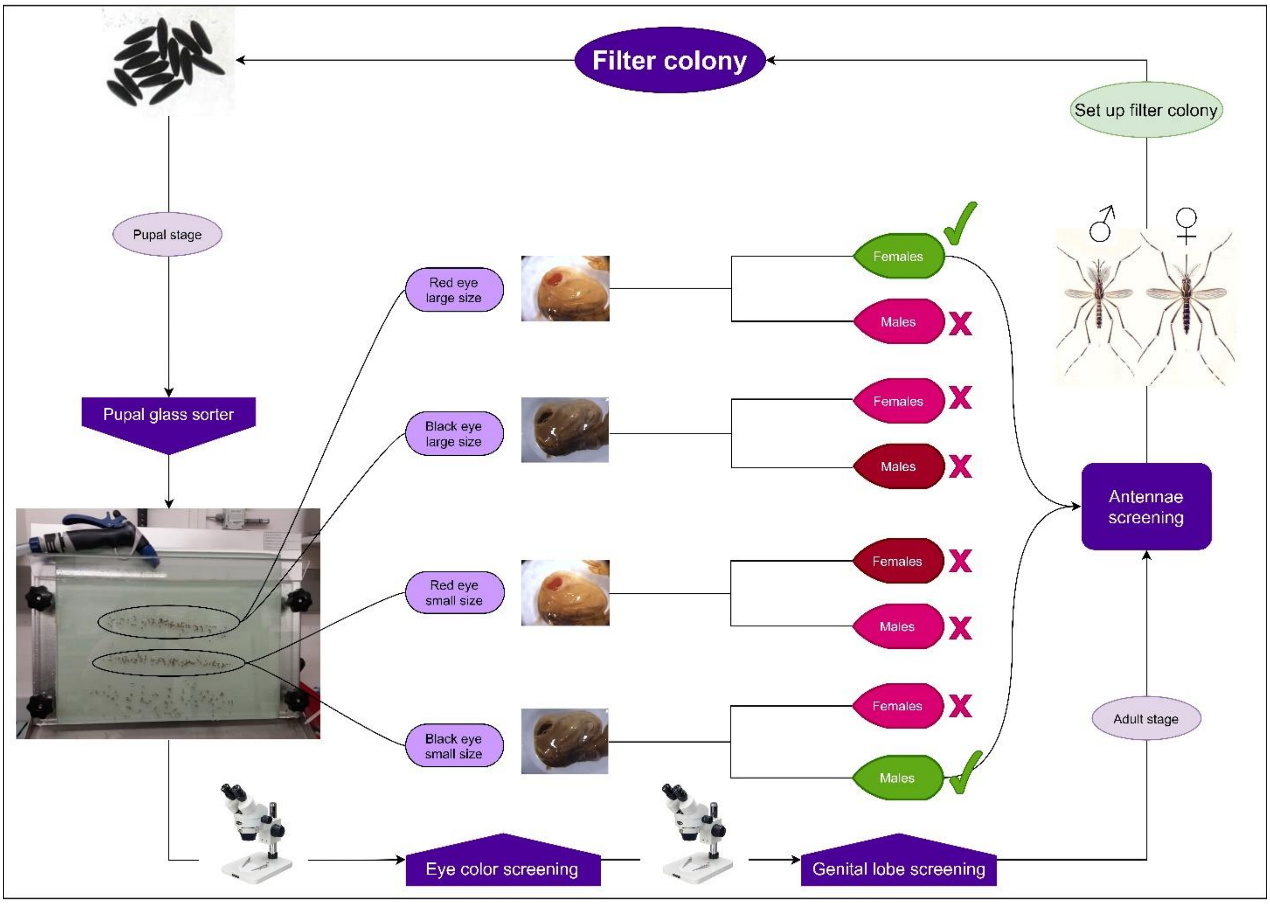

Fig. S1. Development of the filtering system. The filtering system was based on discarding the recombinant offspring during inbreeding. Thus, a GSS was created that consists of heterozygous males with wild-type eyes and homozygous females with mutant eyes (*re* or *w*). The removal of recombinants should be done in each generation using the filter rearing system. Green background color indicates the fractions that were eventually used for the filtered GSS colony. Red background color indicates the recombinant progeny. Sorting was conducted during pupal stage using a glass sorting apparatus[1] which segregated males from females based on pupal size dimorphism (female pupae are larger than male pupae). The two groups of the pupae collected from the sorting procedure were kept separately and subsequently screened under stereomicroscope in respect to their eye color. At this point, four different phenotypes of pupae were recorded: a) large *re* (or we) pupae, b) large pupae with wild-type eyes, c) small *re* (or we) pupae, and d) small pupae with wild-type eyes. The gender of the putative recombinants was verified by screening the pupal genital lobe shape under stereomicroscope to ensure the proper calculation of recombination rates. The offspring were further screened in terms of gender upon emergence in order to compensate any failures in sex separation during pupal sorting with the glass separator, resulting in the following fractions: a) large *re* (or we) pupae - females, b) large pupae with wild-type eyes - females, c) small *re* (or we) pupae - males, d) small pupae with wild-type eyes – males, e) large *re* (or we) pupae – males, f) large pupae with wild-type eyes – males, g) small *re* (or we) pupae – females, h) small pupae with wild-type eyes - females. Additional sex determination of the putative non-recombinants during the adult stage augments the effectiveness of the system by guaranteeing the absence of escapers from both filters (size and eye color) and thus ensuring the filtered colony purity. The recombinant progeny (wild-type females and re or we males) were discarded and only the parental progeny were kept (re or we females and wild-type males).

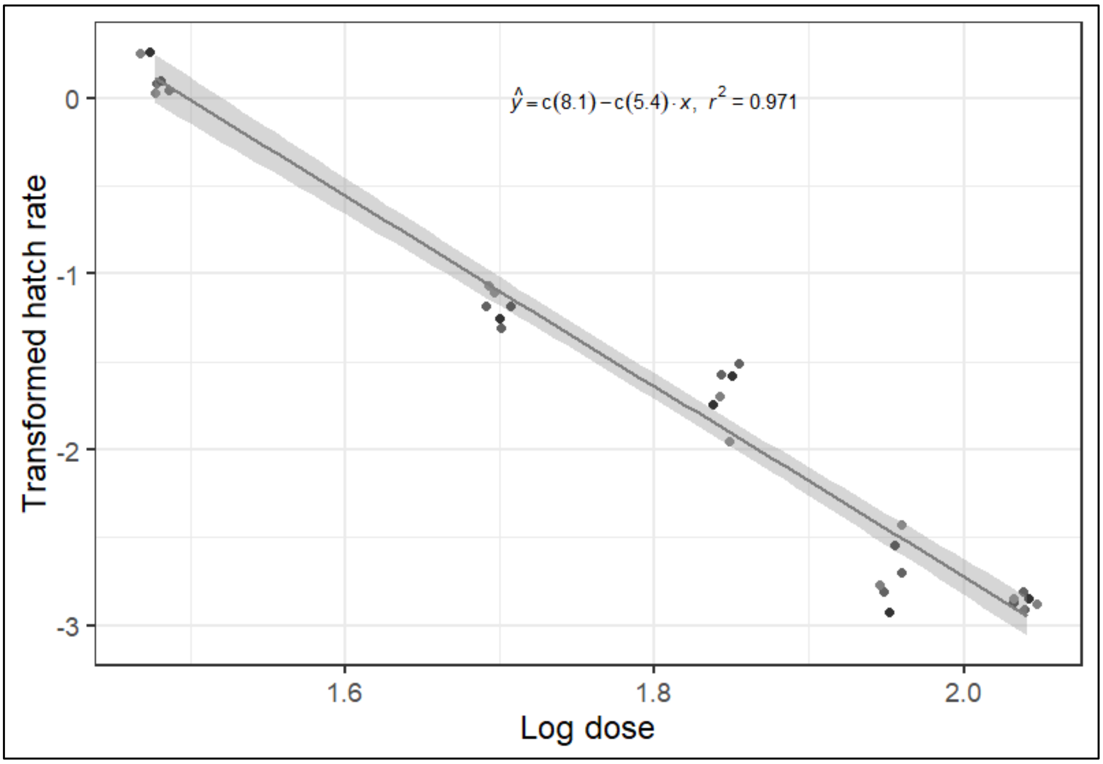

Fig. S2. Radiation dose curve of sterility for the Red-eye GSS males. The radiation dose-response curve was developed to determine the lowest radiation dose able to achieve more than 99.9% male sterility, with the lowest fitness cost. Male pupae 30-36 hours after pupation were gamma-irradiated at 0, 30, 50, 70, 90 and 110 Gy in a Gammacell 220[2]. For each dose there were three technical replicates within two biological repetitions with 30 male pupae each (Figure 2, transformed hatch rate replicates are represented by points and repetitions in grayscale the standard error is shown as gray shaded area). In order to apply the most appropriate dose-response model, the hatch rate and the doses were transformed to obtain the highest r^2 value ($r^2 = 0.97$). The statistical analysis was then performed using a linear regression model (Supplementary Material – Statistical Analysis Report and spreadsheet data). There was significantly statistical difference among the log doses regarding residual fertility (LM F: 289.9, d.f. = 3, 26, $P < 0.05$) The mean hatch rate was 87.6% for 0 Gy, 29.3% for 30 Gy, 5.2% for 50 Gy, 2.3% for 70 Gy, 0.6% for 90 Gy and 0.07% for 110 Gy. Based on this data, 90 Gy is the lower radiation dose to achieve more than 99% sterility and this dose was further used for all irradiation experiments included in this study, except the irradiation-induced recombination suppressors experiment.

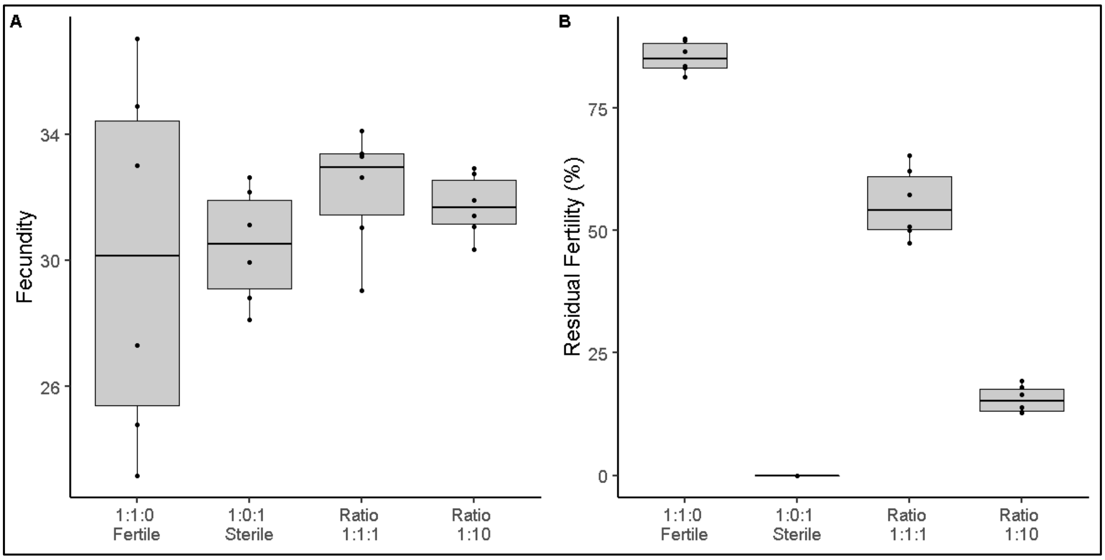

Fig. S3. Male mating competitiveness test. The male mating competitiveness test was performed with two different ratios of [wild type female : wild type male : irradiated Red-eye GSS male] 1:1:10 and 1:1:1. The competitiveness was calculated by the formula $c = N/S * (Hn - Ho)/(Ho - Hs)$, where N is the total number of wild-type males, S is the total number of sterile males, Hn is the wild-type hatch rate ("1:1:0" fertile cage), Hs is the sterile hatch rate ("1:0:1" sterile cage) and the Ho is the observed hatch rate of each ratio ("1:1:1" or "1:1:10"). Regarding the fecundity (number of eggs/female - Figure 3A), there was no statistically significant difference (GLM F= 0.64, d.f.=3, 20, $P > 0.05$) for both ratios tested (1:1:10 and 1:1:1) compared to the "1:0:1" sterile cage and the "1:1:0" fertile cage. The ratios "1:1:1" and "1:1:10" had, on average, 32.2 and 31.7 eggs/female, respectively and the "1:1:0" fertile cage and "1:0:1" sterile cage had, on average, 30 and 30.5 eggs/female respectively.
However, for the fertility (percentage of hatch rate – Figure 3B), the data showed a clear difference among the ratios, the "1:1:0" fertile cage had 85.43% fertility, while the "1:0:1" had 0% fertility. The ratios "1:1:1" and "1:1:10" had 55.5% and 15% fertility, respectively (GLM F=661.8, d.f.=3, 20, $P<0.05$). The calculated Fried Index had a geometric average of 0.53 (SE = 0.08) and 0.44 (SE = 0.04) for the "1:1:1" and "1:1:10" ratio respectively, with an Induced Sterility Index (ISI)[3] based on the geometric mean of 34.3% (SE = 3.19) and 81.7% (SE = 1.34) respectively for "1:1:1" and "1:1:10" ratios, suggesting an appropriate release ratio of "1:1:10".
Box-plot shows the center line as the median of each ratio; the box limits are the interquartile range at 25th and 75th percentile; the lines correspond to the minimum and maximum considering 1.5x correspondent to the quartile range; the points indicate the observed data.

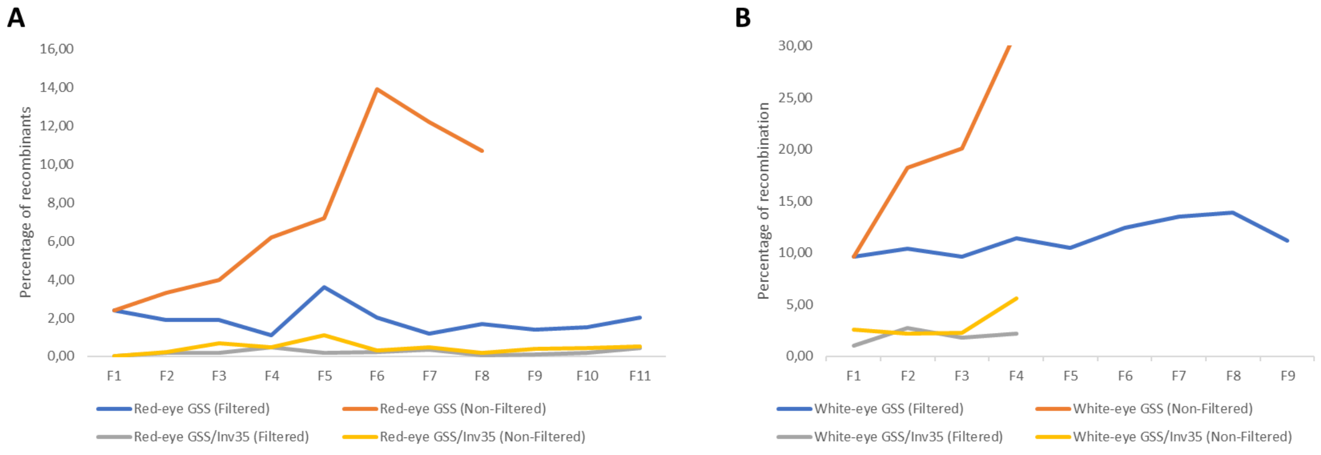

Fig. S4. The effect of the Inv35 on recombination frequencies and accumulation of recombinants under non-filtered conditions. A: filtered and non-filtered colonies of the Red-eye GSS and the Red-eye GSS/Inv35 strains; B: filtered and non-filtered colonies of the White-eye GSS and the White-eye GSS/Inv35 strains

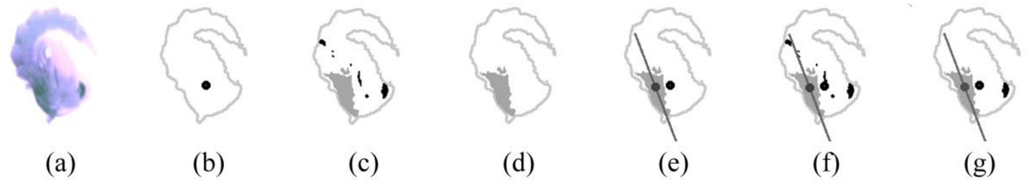

Fig. S5. Identification of the pupal eye and its location. (a) Original pupa with the background removed. (b) Contour and centroid. (c) BLOBS obtained after applying a threshold. (d) The larger area determines the cephalothorax. (e) Adjusted orientation line. (f) Set of BLOBS that could be eyes (without considering the cephalothorax). (g) A BLOB successfully passes all the filters, determining that the pupa corresponds to a male.

More detailed, under a powerful light in the infra-red (IR) spectrum, the melanised eye of an *Aedes* mosquito pupa is seen as a dark BLOB (Binary Large Object) that can be visually distinguished from the rest of the body. Conversely, the red eye of the females of the Red-eye GSS is lacking melanin and therefore is transparent to the IR light. Other parts of the body of the pupa can also form dark BLOBs and be confused with the eye in certain pupa positions. The proper identification of the black eye of the pupa thus requires a morphological analysis. The proposed algorithm for the recognition of black eyes in the pupae in a lateral position combines several operations, starting with the segmentation of the pupa and calculation of the centroid of the silhouette (Fig. S5a and S5b). The dorsal part of the cephalothorax always appears as a dark area with a large size in relation to the size of the body. By applying a threshold operation (Fig. S5c and S5d), this dark area can be segmented, and its contour can be defined (in the examples set shown in Figure 4, we have applied a threshold level of 56%, but this value may vary depending on the intensity of illumination or camera features). The straight line that better fits this dark area is then calculated by least squares (Fig. S5e). This line defines the orientation of the cephalothorax in most of the cases. The eye of the pupae is expected to be found on the same side of the line where the centroid is. At this point, a first filtering is applied to eliminate the BLOBS that may be a good candidate for a black eye but are on the wrong side. The eye of the pupa is always in the opposite part of the back, which has already been identified. A minimum relative distance from the eye to the back can be characterized. At a second filtering step, the minimum distance of each of the BLOBS to the line that defines the orientation is calculated and those who are closer than a certain relative threshold are also discarded. Finally, those BLOBS that are beyond a certain relative distance to the centroid of the pupa are also discarded to eliminate the dark areas that often appear in between the abdominal segments (Fig. S5f). Any BLOB that has not been filtered in the previous steps is considered an eye and the pupa is then sorted as a male (Fig. S5g). In some positions of the pupa where the abdomen is twisted backwards, as is the case in pictures 14, 27 and 47 of Figure 4 the orientation line calculated by least squares does not give a good fit and does not define properly the orientation of the cephalothorax. These cases can be detected by analysing the circularity of the convex polygon enveloping the contour shape of the BLOB corresponding to the dorsal part of the cephalothorax of the pupae after applying the automated threshold. When the circularity index is above 0.80, the orientation of the pupae is better defined by the perpendicular to the line obtained from the centroid of the pupa and the centroid of the dark area of the cephalothorax. The rest of the operations remain the same. The background has been subtracted as a preliminary step of the algorithm. A set of pictures and the proposed algorithm is available in the supporting material. The pictures of the pupae used for this analysis have been taken using a camera model "IDS UI-3080CP-C-GL Rev 2" with no IR filter, a "RICHO FL-CC2514-5M" lens and a source of IR light with a wavelength of 850nm. The application that implements the algorithm has been developed in C++ language, using OpenCV 3.4.2 graphics library and Visual C++ 2017.

Tables S1-S3

Table S1. Genetic crosses performed to verify the genetic linkage and genetic distance between a) *red eye* and M locus and b) *white eye* and M locus

	**Nº families**	**♀ re**	**♀ wt**	**♂ re**	**♂ wt**	**Total progeny**	**Mean recombination frequency (± SEM)**	**χ^2 P-value***
**Parental crosses**								
(*re m*/*re m*) X (+ *M*/+ *m*)	27	0	726	0	793	1519	-	-
(+ *m*/+ *m*) X (*re M*/*re m*)	11	0	279	0	297	576	-	-
**F1 inbreeding**								
(*re m*/+ *m*) X (+ *M*/*re m*)	9	121	121	6	261	509	-	0.98
(+ *m*/*re m*) X (*re M*/+ *m*)	9	1	108	121	209	439	-	0.97
**Backcross**								
(*re m*/*re m*) X (+ *M*/*re m*)	9	230	6	3	242	481	1.9 ± 0.82	0.35**
Massive cross	-	373	6	13	389	781	2.4	-
(*re m*/*re m*) X (*re M*/+ *m*)	12	13	483	338	5	839	2.3 ± 0.61	0.45**
Massive cross	-	3	315	281	5	604	1.3	-
	**Nº families**	**♀ we**	**♀ wt**	**♂ we**	**♂ wt**	**Total progeny**	**Mean recombination frequency (± SEM)**	**χ^2 P-value***
**Parental crosses**								
(*we m*/*we m*) X (+ *M*/+ *m*)	20	0	759	0	797	1556	-	-
(+ *m*/+ *m*) X (*we M*/*we m*)	10	0	169	0	181	350	-	-
**F1 inbreeding**								
(*we m*/+ *m*) X (+ *M*/*we m*)	13	151	221	21	348	741	-	0.99
(+ *m*/*we m*) X (*we M*/+ *m*)	14	53	391	187	322	953	-	0.98
**Backcross**								
(*we m*/*we m*) X (+ *M*/*we m*)	13	317	40	34	326	717	11.35 ± 1.7	0.58**
Massive cross	-	103	13	10	114	240	9.6	-
(*re m*/*re m*) X (*re M*/+ *m*)	11	42	291	247	50	630	14.77 ± 1.3	0.68**
Massive cross		5	49	58	11	123	13.0	-

Results regarding the progeny of the reciprocal crosses between the two mutant strains (Red-eyes, Higgs White-eyes) and the wild-type BRA strain (parental crosses), the F1 inbreeding crosses and test backcrossing. Considering both sex and eye color, there are four phenotypes per backcross, deriving from the four discrete genotypes. For the cross involving the red eye mutation, there are wild type males and red eye females deriving from the respective expected, non-recombinant genotypes (47.8% and 49.8%, respectively) and red eye males plus wild type females, deriving from the respective non-expected, recombinant genotypes (47.8% and 49.8%, respectively). For the cross involving the Higgs white eye mutation, there are wild type males and white eye females deriving from the respective expected, non-recombinant genotypes and white eye males plus wild type females, deriving from the respective non-expected, recombinant genotypes. White-eye females and wild-type males (42.8% and 45.9%, respectively) that were the parental genotypes, and white-eye males and wild-type females (5.5% and 5.9%, respectively) that were the recombinant progeny. Recombination frequencies between the two mutations and the M locus are in the range of those previously described[4–7].

*Chi-square test was used to compare the observed ratio of eye color (wt : re) against the expected ratios of 3 : 1 for F1 inbreeding crosses and the observed sex ratio of mutant progeny against the expected 1 : 1 for Backcrosses.
** Rejected null hypothesis of 1:1 sex ratio in mutant progeny, which indicates sex-linkage.

Table S2. Recombination rate in filtered and non-filtered Red-Eye GSS and White-eye GSS colonies.

Strain	FRS	F	GC	Recombination rate			Number of mosquitoes screened
				total	males	females	
Red-Eye GSS	Yes	1	mix	0.024	0.032	0.016	254
		2	1	0.019	0.012	0.025	481
		3	1	0.019	0.023	0.016	777
			2	0.023	0.024	0.022	3161
		4	1	0.011	0.011	0.013	5518
		5	1	0.036	0.034	0.037	2759
			2	0.038	0.0.39	0.037	807
		6	1	0.020	0.027	0.012	4417
		7	1	0.012	0.010	0.015	728
			2	0.009	0.012	0.007	1524
			3	0.014	0.014	0.014	1594
		8	1	0.017	0.025	0.012	457
			2	0.014	0.012	0.016	208
		9	1	0.014	0.021	0.007	888
			3	0.011	0.011	0.012	785
		10	1	0.015	0.021	0.009	898
			3	0.012	0.011	0.012	785
		11	1	0.020	0.016	0.024	1454
	No	1	1	0.033	0.039	0.027	1558
		2	1	0.040	0.047	0.034	872
		3	1	0.062	0.065	0.058	2028
		4	2	0.072	0.078	0.067	664
		6	1	0.139	0.120	0.160	2073
		7	1	0.122	0.109	0.136	872
		8	1	0.107	0.163	0.057	197
White-Eye GSS	Yes	1	mix	0.096	0.081	0.112	311
		2	1	0.104	0.096	0.112	721
		3	1	0.096	0.081	0.112	240
		4	1	0.114	0.102	0.125	2078
		5	1	0.105	0.107	0.102	468
		6	1	0.124	0.136	0.111	711
		7	1	0.138	0.119	0.157	167
			2	0.135	0.122	0.150	1685
			3	0.128	0.135	0.118	1601
		8	2	0.139	0.135	0.142	714
		9	2	0.112	0.074	0.146	744
	No	1	1	0.182	0.130	0.243	332
		2	1	0.201	0.089	0.335	617
		3	1	0.315	0.195	0.467	1354
		4	1	0.298	0.122	0.517	924
Red-Eye GSS/Inv35	Yes	1	mix	0.0000	0.0000	0.0000	990
		2	mix	0.0018	0.0013	0.0030	2264
		3	mix	0.0020	0.0028	0.0011	1961
		4	mix	0.0046	0.0060	0.0032	2409
		5	mix	0.0020	0	0.0050	1496
		6	mix	0.0021	0.0009	0.0042	1873
		7	mix	0.0037	0.0013	0.0059	1637
		8	mix	0.0006	0.0011	0	1745
		9	mix	0.0011	0.0020	0	904
		10	mix	0.0018	0.0026	0.0011	1713
		11	mix	0.0042	0.0044	0.0040	1433
		12	mix	0.0030	0.0020	0.0040	1852
	No	1	mix	0	0	0	1129
		2	mix	0.0023	0.0026	0.0020	3077
		3	mix	0.0068	0.0046	0.0089	1325
		4	mix	0.0049	0.0014	0.0128	1021

		5	mix	0.0110	0.0120	0.0100	1546
		6	mix	0.0031	0.0042	0.0018	1043
		7	mix	0,.0048	0.0081	0.0016	1238
		8	mix	0.0019	0.0035	0	1583
		9	mix	0.0041	0.0038	0.0047	1220
		10	mix	0.0044	0.0080	0.0024	2022
		11	mix	0.0051	0.0034	0.0071	1579
		12	mix	0.0040	0.0036	0.0042	2277
White-eye GSS/Inv35	Yes	1	mix	0.010	0.0127	0.0073	5197
		2	mix	0.0272	0.0191	0.0358	1430
		3	mix	0.0181	0.0198	0.0168	2481
		4	mix	0.0218	0.0129	0.0318	1602
	No	1	mix	0.0262	0.0354	0.0180	1259
		2	mix	0.0217	0.0224	0.0211	1151
		3	mix	0.0229	0.0166	0.0322	1006
		4	mix	0.0561	0.0560	0.0561	1908

Recombination frequencies were recorded for the two genetic sexing strains (Red-eye GSS and White-eye GSS) for consecutive generations (F) and consecutive gonotrophic cycles (GC) where possible, with and without the filter rearing system (FRS). As evident from the data, the recombination frequency remained constant under filtering conditions, usually between 1.0-2.5% for the Red-eye GSS and 9-13.5% for the White-eye GSS. Without the filtering system, the Red-eye GSS gradually accumulates recombinants, maintaining a partial 'integrity' for few generations (7% after four generations) and then it seems to 'stabilize', having recombinants in the range of 10-13%. The lack of sex bias in the accumulated recombinants evidence that the red eye mutation is not related to severe fitness costs. On the other hand, the White-eye GSS deteriorates rather fast, since the accumulation of recombinant females reaches up to 50% after only four generations. The sex bias in the accumulation of recombinants (more than 50% wild type females *vs* 12.2% white eye males in the fourth generation shows that the white eye mutation is correlated with a severe fitness cost.

We tried to see whether the age of the parents influences recombination frequencies. To address this, recombination frequencies were estimated for consecutive gonotrophic cycles in selected generations. Recombination frequencies were estimated for two gonotrophic cycles of five generations (F3, F5, F8, F9, and F0) and for three gonotrophic cycles of one generation (F7) for the 'Red-Eye GSS' and for three gonotrophic cycles of one generation (F7) for the 'White-Eye GSS'. Our data do not support any parental age effect (positive or negative) on recombination frequencies.

Recombination frequencies were recorded for the Red-eye GSS with the Inv35 (Red-eye GSS/Inv35. Under filtering, recombination remained consistently low, in the range of 0.1-0.2% in twelve consecutive generations. At the same time results from the colony kept without filtering were really encouraging since, after twelve generations, the accumulation of recombinants was still low (less than1%).

Table S3. Quality Control of the BRA wild type, Red-Eye GSS, White-eye GSS, and the Red-eye GSS/Inv35

Quality Control parameters		BRA *(st. dev.)*	'Red-eye' GSS *(st. dev.)*	'White-eye' GSS *(st. dev.)*	'Red-eye' GSS/Inv35 *(st. dev.)*
Egg to L4 recovery (%) (3 x 400 eggs)		73.25^{a} *(3.031)*	76.16^{a} *(8.129)*	60.75^{b} *(2.179)*	49.62^{c} *(5.51)*
Egg to pupa recovery (%) (3 x 400 eggs)		71.66^{a} *(2.516)*	74.00^{a} *(9.148)*	59.58^{b} *(1.664)*	48.87^{c} *(5.57)*
Egg to adult recovery (%) (3 x 400 eggs)		63.08^{a} *(0.877)*	62.91^{a} *(8.281)*	46.16^{b} *(3.502)*	46.53^{b} *(5.55)*
Pupa to adult recovery (%) (3 x 400 eggs)		88.08^{a} *(2.789)*	84.96^{a} *(1.955)*	77.43^{b} *(4.195)*	95.18^{c} *(1.53)*
Hatching (%) (3 x 100 eggs)		nt	43^{a} *(2.645)*	nt	35^{b} *(1.732)*
L1 to pupa recovery (%) (3 x 100L1)		nt	86.33^{a} *(4.509)*	nt	74^{b} *(3.605)*
L1 to adult recovery (%) (3 x 100 L1)		nt	79^{a} *(5.291)*	nt	67^{b} *(3.605)*
Sex ratio (male pupae: total pupae ratio)		0.49 *(0.032)*	0.475 *(0.065)*	0.486 *(0.044)*	0.539 *(0.051)*
Sex ratio (males: total adults' ratio)		0.477 *(0.034)*	0.475 *(0.069)*	0.469 *(0.044)*	0.542 *(0.032)*
Pupal weight (mg -average)	M	1.52 *(0.046)*	1.684 *(0.095)*	1.746 *(0.037)*	2.187 *(0.214)*
	F	2.19 *(0.071)*	1.972 *(0.072)*	3.104 *(0.118)*	3.562 *(0.494)*
Immature development duration (days - average)	M	8.65 *(0.03)*	8.62 *(0.03)*	8.89 *(0.05)*	Not tested
	F	9.32 *(0.03)*	9.16 *(0.03)*	9.79 *(0.06)*	Not tested
Fecundity		32.40^{a} *(8.80)*	56.90^{a,b} *(7.62)*	63.70^{b} *(6.82)*	52.25^{a,b} *(13.89)*
Male flight ability** (% of fliers)	Not irradiated	75.50 *(6.39)*	83.58 *(5.50)*	32.14 *(11.27)*	62.00 *(9.90)*
	Irradiated	33.22 *(7.06)*	57.00 *(5.05)*	18.82 *(9.07)*	36.14 *(9.806)*
Lifespan (days average)	Males	49.69^{a} *(1.57)*	53.19^{c} *(1.77)*	51.940^{b} *(1.44)*	48.3^{a,b}
	Females	28.05^{a} *(1.02)*	43.22^{b} *(1.33)*	34.799$^{a.b}$ *(1.08)*	46.1^{b}
Male mating competitiveness		control	0.53 *(0.08)*	nt	nt

Quality Control was performed incorporating parameters that are important either for mass rearing (recovery, sex ratio, immature development duration, pupal size, and fecundity) or for downstream SIT application (male flight ability, lifespan, and male mating competitiveness). Exponents (letters) indicate homogenous groups. The Red-eye GSS was clearly more robust than the White-Eye GSS when compared to the BRA wild type strain. Regarding recovery, 'strain' is a significant predictor of egg to pupa (F= 5.438, d.f. = 3 and $P < 0.05$), egg to adult (F= 9.361, d.f. = 3 and $P < 0.05$), and pupa to adult (F= 8.097, d.f. = 3 and $P < 0.05$) recovery. Post-Hoc pairwise comparisons show that the White-eye GSS has a significantly reduced recovery compared to both BRA and Red-eye GSS. There is no significant sex ratio bias or difference among the strains when sex ratio was measured either at pupa (F= 0.082, d.f. = 3 and $P > 0.05$) or adult (F= 0.019, d.f. = 3 and $P > 0.05$) stage.

Immature developmental duration was measured for all strains, aiming to detect any underlying negative effects that would lead either to a) slower development, b) reduction of synchronization for males and females, and c) interference with protandry. Although there may be some differences in developmental duration, there is no negative effect on immature development duration, synchronization of male and female pupation and adult emergence or protandry.

Regarding fecundity, 'strain' is a marginally not significant predictor (F = 3.413, d.f. = 2 and $P = 0.055$). Post-Hoc pairwise comparisons show that BRA strain produces significantly fewer eggs than the two GSSs. At the same

time, gonotrophic cycle (correlated also to the age of adults) is not a significant predictor of fecundity (F= 0.144, d.f. = 1 and $P > 0.05$).
Lifespan and flight ability are important parameters for field applications, directly correlated also with the fitness of a strain. Our data show that 'strain' is a significant predictor of male (Chi-Square= 22.801, d.f. = 3 and $P < 0.05$) and female (Chi-Square= 159.413, d.f. = 3 and $P < 0.05$) lifespan and male flight ability. Regarding male lifespan, pairwise Post-Hoc comparisons indicate that Red-eye GSS has significantly higher lifespan than the other two strains. Differences are more severe in the female lifespan. More specifically, BRA females have a significantly lower lifespan than all other females and at the same time White-eye females live significantly less than the Red-eye GSS. In respect to male flight ability, 'strain' (F= 61.059, d.f. = 2 and $P < 0.05$) is a significant predictor. Pairwise Post-Hoc comparisons indicate that the White-eye GSS presents significantly reduced flight ability compared to the other two strains and that the Red-eye GSS has significantly higher flight ability than the wild type BRA. Although the irradiation reduced the flight ability of all strains tested, the Red-eye GSS exhibited significantly higher flight ability than both the wild type BRA and the White-eye GSS (F= 56.488, d.f. = 1 and $P < 0.05$).

The Red-eye GSS with the Inv35 (Red-eye GSS/Inv35) shows significantly lower egg to pupa (F= 22.430, d.f. = 1 and $P < 0.05$) and egg to adult recovery (F= 9.982, d.f. = 1 and $P < 0.05$), but significantly higher pupa to adult (F= 116.406, d.f. = 1 and $P < 0.05$) recovery compared to the original Red-eye GSS. To exclude the effect of reduced hatching, the L1 to pupation recovery was recorded for both strains, showing a significant reduction in the Red-eye GSS/Inv35 strain (F= 18.013, d.f. = 1 and $P < 0.05$) There is a marginally significant difference in sex ratio at pupal (F= 5.781, d.f. =1 and $P = 0.043$) but not at adult (F= 3.976, d.f. = 1 and $P > 0.05$) stage, deriving from both a slight male deficiency in the original strain and a male excess in the Red-eye GSS/Inv35 line. However, the importance (and robustness) of such differences must be evaluated in different generations. Regarding fecundity, there are no significant differences in egg production for two gonotrophic cycles (F= 1.310, d.f. = 1 and $P > 0.05$). In respect to male flight ability it is significantly reduced for the Red-Eye GSS/Inv35 ('strain': F= 26.126, d.f. = 1 and $P < 0.05$). On the other hand, the flight ability of this strain is better than that of the White-eye GSS and comparable to the one of the wild type BRA strain. To further address the origin of the decreased productivity of the Red-eye GSS/Inv35, a smaller experiment was done to directly compare with the Red-eye GSS. Inv 35 was found to have a significant effect in the reduction of hatching (F= 19.2, d.f. = 1 and $P < 0.05$) and downstream productivity as measured from the L1 to pupa recovery (F= 13.690, d.f. = 1 and $P < 0.05$) and L1 to adult recovery (F= 10.537, d.f. = 1 and $P < 0.05$).

References

1. Focks DA. 1980 An improved separator for the developmental stages, sexes, and species of mosquitoes (Diptera: Culicidae). *Journal of medical entomology* **17**, 567–568. (doi:10.1093/jmedent/17.6.567)
2. Helinski MEH, Parker AG, Knols BGJ. 2006 Radiation-induced sterility for pupal and adult stages of the malaria mosquito Anopheles arabiensis. *Malaria Journal* **5**. (doi:10.1186/1475-2875-5-41)
3. Abbott WS. 1925 A Method of Computing the Effectiveness of an Insecticide. *Journal of Economic Entomology* **18**, 265–267.
4. Bhalla SC, Craig Jr. GB. 1970 Linkage analysis of chromosome 1 of _Aedes aegypti_. *Canadian Journal of Genetics and Cytology* **12**, 425–435.
5. Bhalla SC. 1968 White eye, a new sex-linked mutant of Aedes aegypti. *Mosquito News.* , 380–385.
6. Dickson LB, Sharakhova M V, Timoshevskiy VA, Fleming KL, Caspary A, Sylla M, Black WC. 2016 Senegalese Aedes aegypti (L) Is Associated with Chromosome Rearrangements. *PLOS Neglected Tropical Diseases* **10**, 1–28. (doi:10.1371/journal.pntd.0004626)
7. Ouda NA, Wood RJ. 1985 Variation in recombination M/m-re in three strains of mosquito _Aedes aegypti_ (L.). *Journal of Biological Sciences Research* **16**, 153–173.
