# Supplementary material for: Genetic sexing strains for the population suppression of the mosquito vector Aedes aegypti
Source: Philos Trans R Soc Lond B Biol Sci. 2020 Dec 28;376(1818):20190808. doi: 10.1098/rstb.2019.0808 (PMC7776939; doi:10.1098/rstb.2019.0808)

Supplementary material

Electronic Supplementary Material 2: Statistical Report for the Irradiation Studies using the *Ae. aegypti* Red-eyes GSS

Libraries and Formulas

```
library(tidyverse) #graphs, figures and pipe function
library(rcompanion) #compareGLM()
library(EnvStats) #geometric average (geoMean) for fried index
library(ggpubr) #ggarrange()

#Customized formulas

##Standard Error (SE)
se.function<-function(x){
  sd(x)/sqrt(length(x))
}

##Basic statics function for each variable
multi.fun <- function(x) {
      c(min = min(x), mean = mean(x), max=max(x), sd = sd(x), se=se.function(x))
}

##Function to obtain and plot linear regression formula
linear <- function(k) {
  z <- list(xx = format(coef(k)[1], digits = 2),
            yy = format(abs(coef(k)[2]), digits = 2),
            r2 = format(summary(k)$r.squared, digits = 3));
  if (coef(k)[2] >= 0)  {
    eq <- substitute(italic(hat(y)) == xx + yy %.% italic(x)*","~~italic(r)^2~"="~r2,z)
  } else {
    eq <- substitute(italic(hat(y)) == xx - yy %.% italic(x)*","~~italic(r)^2~"="~r2,z)
  }
  as.character(as.expression(eq));
}
```

Article I. Male Competitiveness for the Red-eye GSS- Summary

```
#data bank and spliting data for calculations
male_comp<-read.csv(params$male_comp)
male_comp_new<-by(male_comp, male_comp$ratio, list)

#Means and standards errors
stats_eggs<-round(sapply(split(male_comp$n_eggs_female, male_comp$ratio), multi.fun),2)
stats_hatch<-round(sapply(split(male_comp$n_larvae/male_comp$n_eggs, male_comp$ratio), mult
i.fun),2)
```

Table 1 | Male Mating Competitiveness for the Red-eye GSS Summary Data

Ratio	Mean Fecundity	Mean Fertility (%)
1:1	32.25 (± 0.77)	0.56 (± 0.03)
1:10	31.74 (± 0.41)	0.16 (± 0.01)
CTRL-ST	30.47 (± 0.74)	0 (± 0)
CTRL-WT	30.03 (± 2.34)	0.85 (± 0.01)

Values in the parentheses correspond to the standard error of the mean.

(a) ***Fecundity***

```
glm_red_eggs<-glm(n_eggs_female~ratio, data=male_comp, family = poisson(link="log"))
summary(glm_red_eggs)
```

```
##
## Call:
## glm(formula = n_eggs_female ~ ratio, family = poisson(link = "log"),
##     data = male_comp)
##
## Deviance Residuals:
##      Min        1Q    Median        3Q       Max
## -1.30424  -0.26383   0.04823   0.23270   1.23126
##
## Coefficients:
##              Estimate Std. Error z value Pr(>|z|)
## (Intercept)   3.47362    0.07188  48.322   <2e-16 ***
## ratio1_10    -0.01617    0.10207  -0.158    0.874
## ratioCTRL_ST -0.05692    0.10314  -0.552    0.581
## ratioCTRL_WT -0.07139    0.10352  -0.690    0.490
## ---
## Signif. codes:  0 '***' 0.001 '**' 0.01 '*' 0.05 '.' 0.1 ' ' 1
##
## (Dispersion parameter for poisson family taken to be 1)
##
##     Null deviance: 7.3827  on 23  degrees of freedom
## Residual deviance: 6.7516  on 20  degrees of freedom
## AIC: Inf
##
## Number of Fisher Scoring iterations: 4
```

```
summary(aov(glm_red_eggs))
```

```
##             Df Sum Sq Mean Sq F value Pr(>F)
## ratio        3  19.64   6.547   0.645  0.595
## Residuals   20 203.00  10.150
```

As demonstrated above, there are no difference on the number of eggs per female (fecundity) among all ratios.

(b) ***Fertility***

```
glm_red_hatch<-glm(n_larvae/n_eggs~ratio, weights = n_eggs, data=male_comp,
                   family=binomial(link = "probit"))
summary(glm_red_hatch)
```

```
##
## Call:
## glm(formula = n_larvae/n_eggs ~ ratio, family = binomial(link = "probit"),
##     data = male_comp, weights = n_eggs)
##
## Deviance Residuals:
##     Min       1Q   Median       3Q      Max
## -6.4067  -1.7360  -0.0005   1.5589   7.8003
##
```

```
## Coefficients:
##               Estimate Std. Error z value Pr(>|z|)
## (Intercept)    0.13120    0.01278  10.265   <2e-16 ***
## ratio1_10     -1.14307    0.03421 -33.415   <2e-16 ***
## ratioCTRL_ST  -6.59738  112.40013  -0.059    0.953
## ratioCTRL_WT   0.93067    0.01845  50.436   <2e-16 ***
## ---
## Signif. codes:  0 '***' 0.001 '**' 0.01 '*' 0.05 '.' 0.1 ' ' 1
##
## (Dispersion parameter for binomial family taken to be 1)
##
##     Null deviance: 27604.2  on 23  degrees of freedom
## Residual deviance:   267.6  on 20  degrees of freedom
## AIC: 401.5
##
## Number of Fisher Scoring iterations: 15
```

```
summary(aov(glm_red_hatch))
```

```
##             Df Sum Sq Mean Sq F value Pr(>F)
## ratio        3   5298  1765.9   661.8 <2e-16 ***
## Residuals   20     53     2.7
## ---
## Signif. codes:  0 '***' 0.001 '**' 0.01 '*' 0.05 '.' 0.1 ' ' 1
```

As demonstrated above, there are difference on the hatch rate (fertility) among all ratios.

Figure 1 | Fecundity and fertility of each cross type and ratio

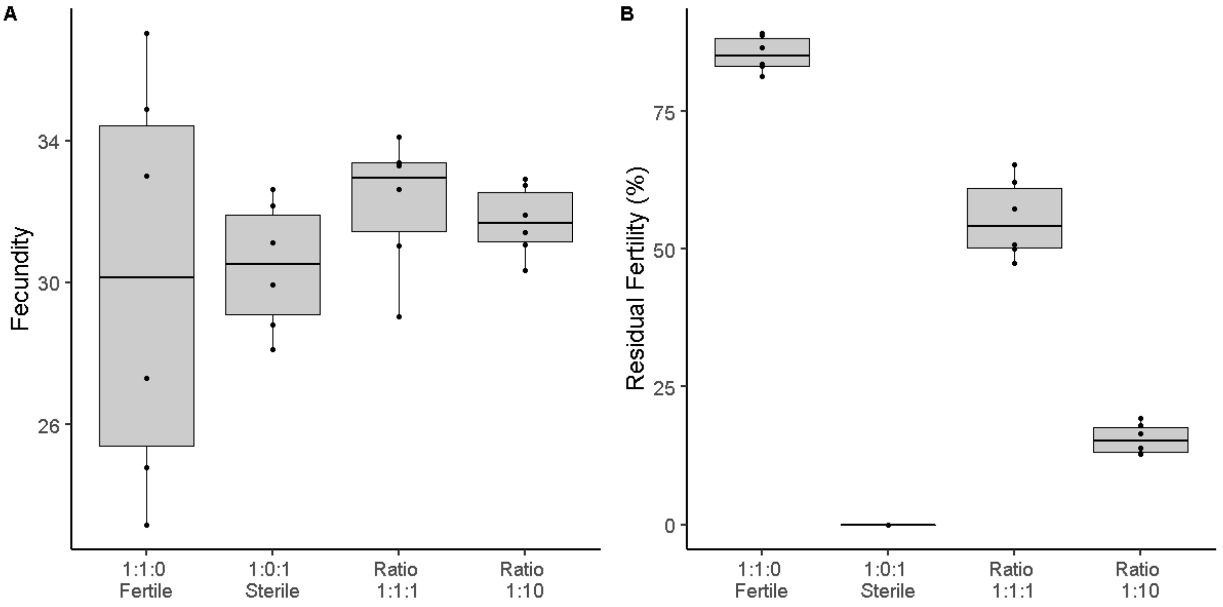

Box-plot shows the center line as the median of each ratio, the box limits is the interquartile range at 25th and 75th percentile, the lines correspond to the minimum and maximum considering 1.5x correspondent to the quartile range; the points indicate the observed data.

Section 1.02 ***Male Mating Competitiveness - Fried Index (C) for the Red-eye GSS***

```
#Loading and splitting data bank
male_comp<-read.csv(params$male_comp)
male_comp_new<-by(male_comp, male_comp$ratio, list)

#Calculation of the competitiveness ratio 1:1
c_1_1<-((male_comp_new$CTRL_WT[,13]-male_comp_new$`1_1`[,13])/
      (male_comp_new$`1_1`[,13]-male_comp_new$CTRL_ST[,13]))*
      (male_comp_new$`1_1`[,7]/male_comp_new$`1_1`[,8])

gmean_1_1<-round(Gmean(c_1_1),2)

SEM_1_1<-round(sd(c_1_1, na.rm = TRUE)/sqrt(length(c_1_1)),2)

#Calculation of the competitiveness ratio 1:10
c_1_10<-((male_comp_new$CTRL_WT[,13]-male_comp_new$`1_10`[,13])/
       (male_comp_new$`1_10`[,13]-male_comp_new$CTRL_ST[,13]))*
       (male_comp_new$`1_10`[,7]/male_comp_new$`1_10`[,8])

gmean_1_10<-round(Gmean(c_1_10),2)

SEM_1_10<-round(sd(c_1_10, na.rm = TRUE)/sqrt(length(c_1_10)),2)
```

In relation to the male competitiveness Fried index, the ratio 1:1:1 had a geometric mean of 0.53 with a standard error = 0.08. And the ratio 1:1:10 had a geometric mean of 0.44 with a standard error = 0.04.

Article II. **Induced Sterility Index (ISI) for the Red-eye GSS**

```
#ratio 1:1
is_ratio_1<-(1-(male_comp_new$`1_1`[,13]/male_comp_new$CTRL_WT[,13]))*100
gmean_is_1<-round(Gmean(is_ratio_1),2)
SEM_is_1<-round(sd(is_ratio_1)/sqrt(length(is_ratio_1)),2)
```

```
#ratio 1:10
is_ratio_10<-(1-(male_comp_new$`1_10`[,13]/male_comp_new$CTRL_WT[,13]))*100
gmean_is_10<-round(Gmean(is_ratio_10),2)
SEM_is_10<-round(sd(is_ratio_10)/sqrt(length(is_ratio_10)),2)
```

In relation to the induced sterility index, the ratio 1:1:1 had a geometric mean of 34.3 with a standard error = 3.19. And the ratio 1:1:10 had a geometric mean of 81.7 with a standard error = 1.34.

Table 2 | Fried Index (C) and Induced Sterility Index for the Red-eye GSS

Ratio	Fried Index (C)	Induced Sterility Index
1:1	0.53 (±0.08)	34.3 (±3.19)
1:10	0.44 (±0.04)	81.7 (±1.34)

Values presented as the geometric mean and in the parentheses they correspond to the standard errors.

Article III. Radiation Dose-Response Curve (normalization) for the Red-eye GSS

```
#NED transformation was conducted from the corrected hatch rate, where the control dose (0 Gy) w
as used as base to estimate the hatch rate for each dose. The corrected hatch rate was then transform
ed using the NED (Probit) calculation: inverse standard normal cumulative distribution of the correc
ted hatch rate (excel formula: NORM.S.INV(corrected hatch rate))

male_dose_n<-read.csv(params$male_dose_normal)

#NED transformation linear model
lm_hatch_dose_red_ned<-lm(NED~rad_dose, data=male_dose_n)
summary(lm_hatch_dose_red_ned)

##
## Call:
## lm(formula = NED ~ rad_dose, data = male_dose_n)
##
## Residuals:
##      Min       1Q   Median       3Q      Max
## -0.51825 -0.27814  0.02742  0.25315  0.42401
##
## Coefficients:
##              Estimate Std. Error t value Pr(>|t|)
## (Intercept)  0.966660   0.142515   6.783 2.29e-07 ***
## rad_dose    -0.037481   0.001888 -19.856  < 2e-16 ***
## ---
## Signif. codes:  0 '***' 0.001 '**' 0.01 '*' 0.05 '.' 0.1 ' ' 1
##
## Residual standard error: 0.2924 on 28 degrees of freedom
## Multiple R-squared:  0.9337, Adjusted R-squared:  0.9313
## F-statistic: 394.3 on 1 and 28 DF,  p-value: < 2.2e-16

summary(aov(lm_hatch_dose_red_ned))

##             Df Sum Sq Mean Sq F value Pr(>F)
## rad_dose     1  33.72   33.72   394.3 <2e-16 ***
## Residuals   28   2.39    0.09
```

```
## ---
## Signif. codes:  0 '***' 0.001 '**' 0.01 '*' 0.05 '.' 0.1 ' ' 1
```

```r
#NED transformation + log(dose) linear model
lm_hatch_log_dose_red_ned<-lm(NED~log_dose, data=male_dose_n)
summary(lm_hatch_log_dose_red_ned)
```

```
##
## Call:
## lm(formula = NED ~ log_dose, data = male_dose_n)
##
## Residuals:
##      Min       1Q   Median       3Q      Max
## -0.44926 -0.08983  0.00641  0.12961  0.37605
##
## Coefficients:
##             Estimate Std. Error t value Pr(>|t|)
## (Intercept)   8.1274     0.3220   25.24   <2e-16 ***
## log_dose     -5.4257     0.1775  -30.57   <2e-16 ***
## ---
## Signif. codes:  0 '***' 0.001 '**' 0.01 '*' 0.05 '.' 0.1 ' ' 1
##
## Residual standard error: 0.1937 on 28 degrees of freedom
## Multiple R-squared:  0.9709, Adjusted R-squared:  0.9699
## F-statistic: 934.4 on 1 and 28 DF,  p-value: < 2.2e-16
```

```r
summary(aov(lm_hatch_log_dose_red_ned))
```

```
##             Df Sum Sq Mean Sq F value Pr(>F)
## log_dose     1  35.06   35.06   934.4 <2e-16 ***
## Residuals   28   1.05    0.04
## ---
## Signif. codes:  0 '***' 0.001 '**' 0.01 '*' 0.05 '.' 0.1 ' ' 1
```

Regarding the two models above, using NED transformation, the highest r^2 is the one also presenting the log transformation of the doses (r^2=0.97). In this linear model there is statistical difference among the log(doses) with the aproximatedly anti-log(dose) of 90 Gy the lowest dose to promote more than 95% sterility.

Figure 2 | Dose-Response Curve for Fertility with normalization for the Red-eye GSS

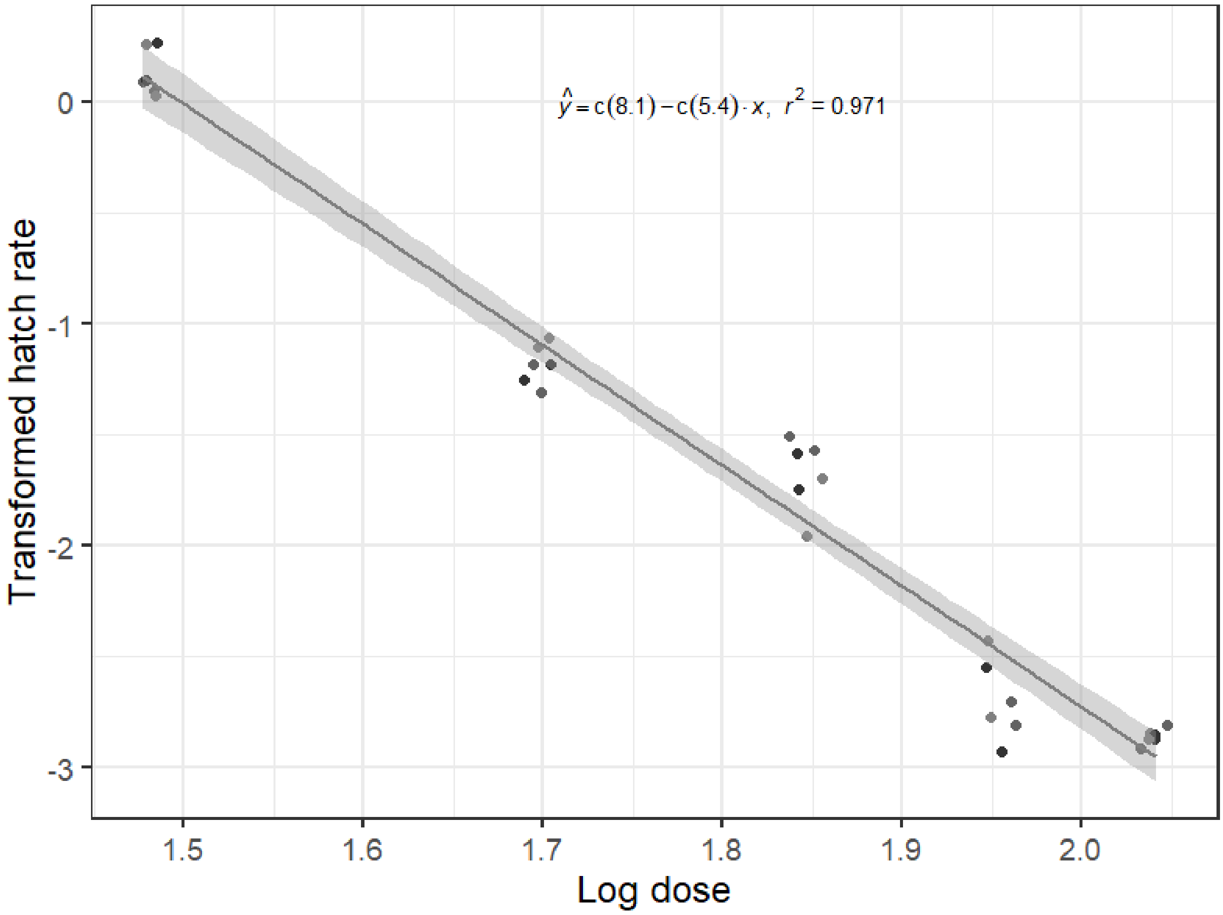

Transformed fertility model in which the replicates are represented by points. Two biological replicates are shown, one in dark grey and the other in light gray color. The gray shaded area depicts the standard error.

(a) **Dose-response for the Red-eye GSS - Summary**

```
male_dose<-read.csv(params$male_dose)

stats_eggs_dose<-sapply(split(male_dose$eggs_female, male_dose$rad_dose), multi.fun) #apply the mean function for each dose
round(stats_eggs_dose,2)

##          0    30    50    70    90   110
## min  19.67 19.30 15.77 15.47 15.47 17.50
## mean 28.11 27.97 29.17 29.23 27.96 27.31
## max  37.23 40.30 43.87 40.40 38.40 40.60
## sd    6.03  7.04  8.28  8.45  8.03  7.50
## se    1.56  1.82  2.14  2.18  2.07  1.94

stats_hatch_dose<-sapply(split(male_dose$n_larva.1/male_dose$n_eggs*100, male_dose$rad_dose)
, multi.fun) #apply the mean function for each dose
round(stats_hatch_dose,2)

##          0    30    50   70   90  110
## min  74.43  7.13  1.12 0.10 0.09 0.09
## mean 87.74 29.48  5.37 2.45 0.76 0.20
## max  99.73 53.69 12.28 5.82 5.49 0.96
## sd    8.01 18.00  3.90 1.66 1.46 0.22
## se    2.07  4.65  1.01 0.43 0.38 0.06
```

Cage Population Suppression for the Red-eye GSS - Summary

```
cage_supp<-read.csv(params$cage_supp)
stats_hatch_cage_supp<-sapply(split(cage_supp$hatch_rate*100, cage_supp$cross), multi.fun)
round(stats_hatch_cage_supp, 2)
##        1_1  1_10  CTRL
## min  50.19  0.00 78.32
## mean 57.16  2.90 86.90
## max  64.65 13.26 93.09
## sd    4.37  3.60  4.36
## se    1.03  0.85  1.03
```

Cage Population Suppression for the Red-eye GSS - Generalized Linear Model

```
cage_supp_hatch<-glm(n_larvae/n_eggs~cross+week,weights = n_eggs, data=cage_supp, family=b
inomial(link="logit"))
summary(cage_supp_hatch)
##
## Call:
## glm(formula = n_larvae/n_eggs ~ cross + week, family = binomial(link = "logit"),
##     data = cage_supp, weights = n_eggs)
##
## Deviance Residuals:
##     Min       1Q   Median       3Q      Max
## -7.5642  -2.2430  -0.5707   2.0179   6.7618
##
## Coefficients:
##              Estimate Std. Error z value Pr(>|z|)
## (Intercept)  0.266338   0.028954   9.199   <2e-16 ***
## cross1_10   -3.217856   0.078556 -40.963   <2e-16 ***
## crossCTRL    1.601016   0.026378  60.695   <2e-16 ***
## week         0.006063   0.007294   0.831    0.406
## ---
## Signif. codes:  0 '***' 0.001 '**' 0.01 '*' 0.05 '.' 0.1 ' ' 1
##
## (Dispersion parameter for binomial family taken to be 1)
##
##     Null deviance: 11461.62  on 49  degrees of freedom
## Residual deviance:   511.37  on 46  degrees of freedom
##   (4 observations deleted due to missingness)
## AIC: 814.73
##
```

```
## Number of Fisher Scoring iterations: 4
summary(aov(cage_supp_hatch))
##              Df Sum Sq Mean Sq F value Pr(>F)
## cross         2 2289.6  1144.8 769.220 <2e-16 ***
## week          1    0.1     0.1   0.078  0.781
## Residuals    46   68.5     1.5
## ---
## Signif. codes:  0 '***' 0.001 '**' 0.01 '*' 0.05 '.' 0.1 ' ' 1
## 4 observations deleted due to missingness
```

Figure 3 | Cage Population Suppression for the Red-eye GSS

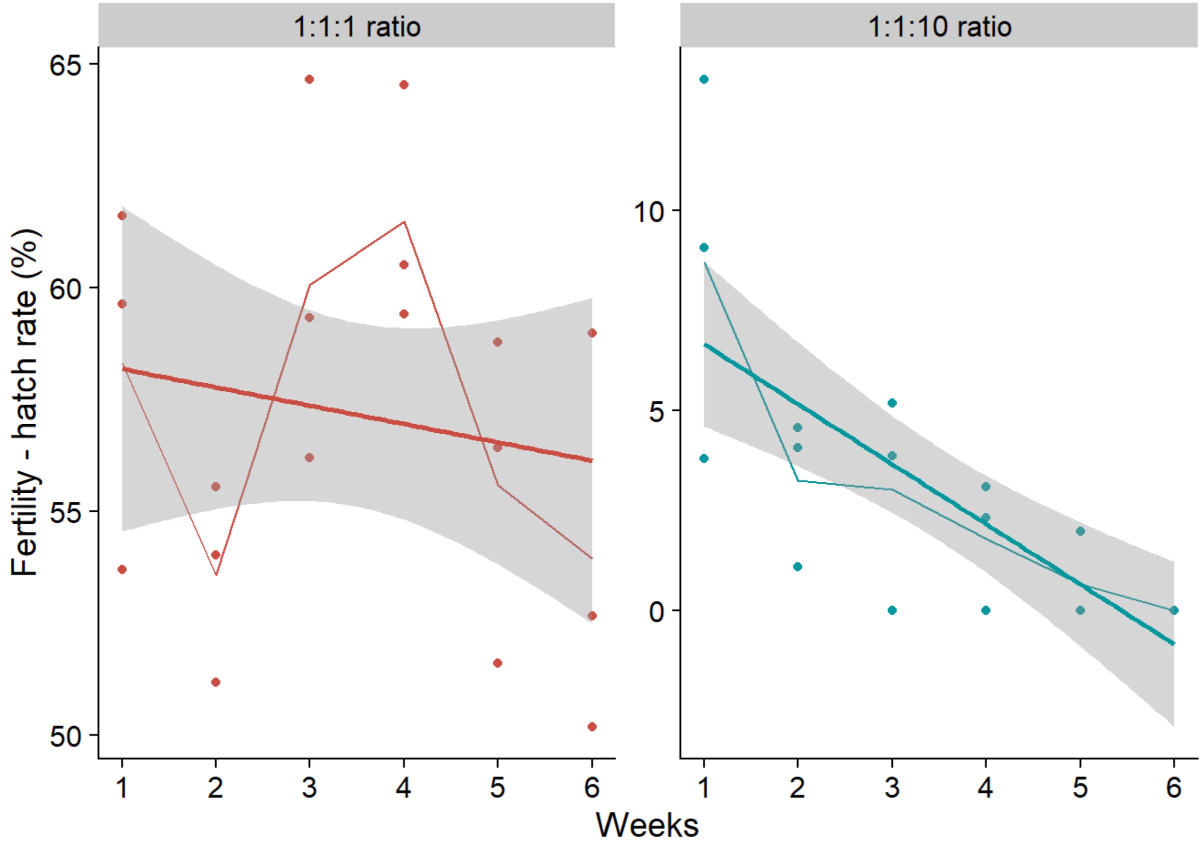

Cage Population Suppression for the Red-eye GSS/Inv35 - Summary

```
cage_supp2<-read.csv(params$cage_supp2)
stats_hatch_cage_supp2<-sapply(split(cage_supp2$hatch_rate*100, cage_supp2$cross), multi.f
un)
round(stats_hatch_cage_supp2, 2)
##        1_1 1_10 CTRL ST CTRL WT
## min  35.14 0.00    0.52   64.39
## mean 42.30 2.38    0.93   70.98
## max  51.19 5.88    1.44   76.97
## sd    3.94 2.05    0.28    2.87
## se    0.76 0.39    0.05    0.55
```

Cage Population Suppression for Red-eye GSS/Inv35 - Generalized Linear Model

```
cage_supp2_hatch<-glm(n_larvae/n_eggs~cross+week,weights = n_eggs, data=cage_supp2, family
=binomial(link="logit"))

summary(cage_supp2_hatch)

##

## Call:

## glm(formula = n_larvae/n_eggs ~ cross + week, family = binomial(link = "logit"),

##     data = cage_supp2, weights = n_eggs)

##

## Deviance Residuals:

##     Min       1Q   Median       3Q      Max

## -9.3851  -2.1416   0.0232   1.6691   9.9852

##

## Coefficients:

##               Estimate Std. Error  z value Pr(>|z|)

## (Intercept)  -0.293747   0.011516  -25.509   <2e-16 ***

## cross1_10    -2.982641   0.058701  -50.811   <2e-16 ***

## crossCTRL ST -4.363659   0.032474 -134.372   <2e-16 ***

## crossCTRL WT  1.204742   0.009413  127.980   <2e-16 ***

## week         -0.003422   0.001825   -1.875   0.0608 .

## ---

## Signif. codes:  0 '***' 0.001 '**' 0.01 '*' 0.05 '.' 0.1 ' ' 1

##

## (Dispersion parameter for binomial family taken to be 1)

##

##     Null deviance: 150499.7  on 107  degrees of freedom

## Residual deviance:   1160.3  on 103  degrees of freedom

## AIC: 1862.8

##

## Number of Fisher Scoring iterations: 4

summary(aov(cage_supp2_hatch))

##              Df Sum Sq Mean Sq  F value Pr(>F)

## cross         3  28179    9393 4257.008 <2e-16 ***

## week          1      1       1    0.233   0.63

## Residuals   103    227       2

## ---

## Signif. codes:  0 '***' 0.001 '**' 0.01 '*' 0.05 '.' 0.1 ' ' 1
```

Figure 4 | Cage Population Suppression for Red-eye GSS/Inv35

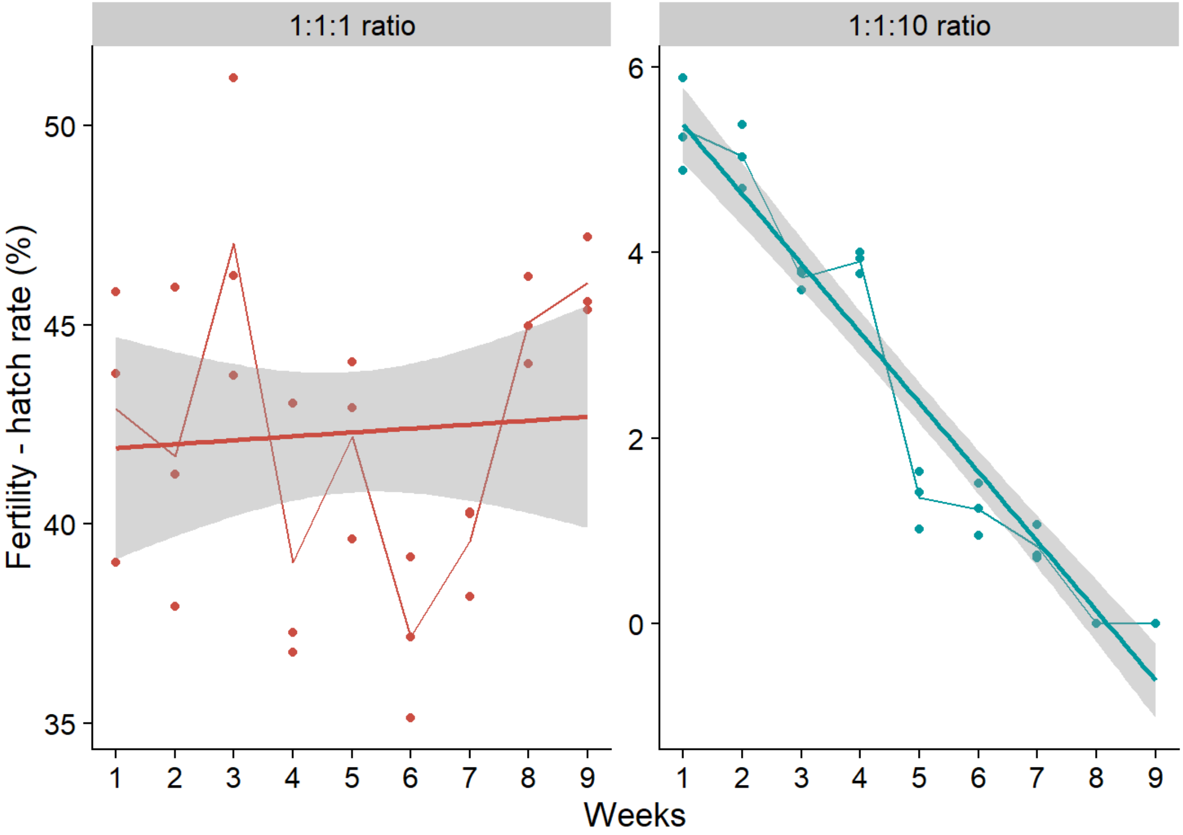

Supplement: Statistical Report for the Irradiation Studies using the Ae. aegypti Red-eyes GSS [file rstb20190808supp2.pdf]
