# Supplementary material for: Genetic sexing strains for the population suppression of the mosquito vector Aedes aegypti
Source: Philos Trans R Soc Lond B Biol Sci. 2020 Dec 28;376(1818):20190808. doi: 10.1098/rstb.2019.0808 (PMC7776939; doi:10.1098/rstb.2019.0808)
Supplement: Detection of the Red Eye Marker for Sex Identification of Aedes Pupae [file rstb20190808supp3.pdf]

Supplementary material

Electronic Supplementary Material 3: Detection of the Red Eye Marker for Sex Identification of Aedes Pupae

```
// -----------------------------------------------------------------------
// "Detection of Red Eye Marker for Sex Identification of Aedes Pupae"
//
// Gustavo Salvador-Herranz          04/12/2019
//
// Visual C++ 2017
// OpenCV 3.4.2
// -----------------------------------------------------------------------

#include "stdafx.h"
#include <opencv2/opencv.hpp>
#include "opencv/cv.h"

#define IMAGE_SIZE 200                              // Size in pixels of the pupae sample image (one
side of a square image)

#define CIRCULARITY_LIMIT 0.8                       // Maximum circularity of the convex polygon
that surrounds
// the dark area of the cephalotorax allowed to adjust a line that characterizes it

#define CONTOUR_THRESHOLD 140                               // Threshold applied to obtain the binary
image corresponding to the pupae_contour

#define DARK_CEPHALOTHORAX_THRESHOLD 200  // Threshold applied to obtain the binary
image corresponding to the dark area
// of the cephalothorax

#define PUPAE_CENTROID_THRESHOLD 230              // Threshold applied to obtain the
centroid of the pupae

#define LINE_EXTENSION 70.0                         // Line length extensión factor, for results
drawing

#define EYE_CANDIDATES_THRESHOLD 220              // Threshold applied to obtain the binary
image corresponding to the eye candidates

#define MIN_DISTANCE_TO_LINE 25                     // Minimum distance from a BLOB to
calculated line to be considered as an eye candidate

#define MAX_DISTANCE_TO_LINE 62                     // Maximum distance from a BLOB to
calculated line to be considered as an eye candidate

#define MAX_DISTANCE_TO_CENTROID 65               // Maximum distance from a BLOB to
calulated centroid to be considered as an eye candidate

#deine MINIMUM_EYE_SIZE 10                        // Minimum BLOB size to be considered as an
eye candidate

using namespace cv;
using namespace std;
```

```
Mat buffer_0;
Mat res_1;
Mat res_2;
Mat male_symbol;
Mat female_symbol;
Mat mosaic_1(IMAGE_SIZE * 5+1 IMAGE_SIZE  * 10 + 1, CV_8UC3, Scalar(255, 255, 255));
Mat mosaic_2(IMAGE_SIZE * 5 + 1, IMAGE_SIZE * 10 + 1, CV_8UC3, Scalar(255, 255, 255));

int current_pupae = 1;
int x = 0;
int y = 0;
int col = 0;

void process_image() {

Mat bgr[3];
	Mat binary_g;
	Mat distance;
	Mat distance_bin;
	Mat distance_n;

	split(buffer_0, bgr);

// Finding pupae external contour -------------------

	threshold(bgr[1], binary_g, CONTOUR_THRESHOLD, 255,
CV_THRESH_BINARY_INV);
	vector<vector<Point>> contours;
	Mat contourOutput = binary_g.clone();
	findContours(contourOutput, contours, CV_RETR_LIST, CV_CHAIN_APPROX_NONE);
	double largest_area = 0;
	int largest_contour_index = 0;
	for (int idx = 0; idx < contours.size(); idx++) {
		double a = contourArea(contours[idx], false);
		if (a > largest_area) {
			largest_area = a;
			largest_contour_index = idx;
		}
	}

	Mat pupae_contour = binary_g.clone();

	pupae_contour.setTo(Scalar(0, 0, 0));
	drawContours(pupae_contour, contours, largest_contour_index, Scalar(255, 255, 255), -1);
	drawContours(res_2, contours, largest_contour_index, Scalar(200, 200, 200), 2);
	Mat pupae_contour_2 = pupae_contour.clone();

// Finding pupae centroid -------------------

	distanceTransform(binary_g, distance, CV_DIST_L2, 3);
	normalize(distance, distance_n, 0, 1.0, NORM_MINMAX);
	distance_n.convertTo(distance_bin, CV_8U, 255);
```

```
	threshold(distance_bin, distance_bin, PUPAE_CENTROID_THRESHOLD, 255,
CV_THRESH_BINARY);

	vector<vector<Point>> centroid_contours;

	findContours(distance_bin, centroid_contours, CV_RETR_LIST,
CV_CHAIN_APPROX_NONE);
	largest_area = 0;
	int largest_centroid_contour_index = 0;
	for (int idx = 0; idx < centroid_contours.size(); idx++) {
		double a = contourArea(centroid_contours[idx], false);
		if (a > largest_area) {
			largest_area = a;
			largest_centroid_contour_index = idx;
		}
	}

	vector<Moments> mu(centroid_contours.size());
	for (int i = 0; i < centroid_contours.size(); i++)
	{
		mu[i] = moments(centroid_contours[i], false);
	}

	CvPoint centroid =	cvPoint((int)(mu[largest_centroid_contour_index].m10 /
mu[largest_centroid_contour_index].m00), (int)(mu[largest_centroid_contour_index].m01 /
			mu[largest_centroid_contour_index].m00));
	circle(pupae_contour, centroid, 3, cvScalar(155, 155, 155), 3, 3, 0);

// Finding pupae biggest dark cephalotorax area -------------------

	Mat dark_cephalothorax;

	threshold(bgr[0], dark_cephalothorax, DARK_CEPHALOTHORAX_THRESHOLD, 255,
CV_THRESH_BINARY_INV);

	vector<vector<Point>> dark_cephalothorax_contours;
	findContours(dark_cephalothorax, dark_cephalothorax_contours, CV_RETR_LIST,
CV_CHAIN_APPROX_NONE);
	largest_area = 0;
	int dark_cephalotorax_largest_contour_index = 0;
	for (int idx = 0; idx < dark_cephalothorax_contours.size(); idx++) {
		double a = contourArea(dark_cephalothorax_contours[idx], false);
		if (a > largest_area) {
			largest_area = a;
			dark_cephalotorax_largest_contour_index = idx;
		}
	}

	dark_cephalothorax.setTo(Scalar(0, 0, 0));
	drawContours(res_2, dark_cephalothorax_contours,
dark_cephalotorax_largest_contour_index, Scalar(200, 200, 200), -1);
	circle(res_2, centroid, 3, cvScalar(0, 0, 0), 3, 3, 0);

// Finding the centroid of the dark part of the cephalothorax ---------------
```

```
	vector<Moments> mu_dark(dark_cephalothorax_contours.size());
	for (int i = 0; i < dark_cephalothorax_contours.size(); i++)
	{
		mu_dark[i] = moments(dark_cephalothorax_contours[i], false);
	}

	CvPoint centroid_dark = 
cvPoint((int)(mu_dark[dark_cephalotorax_largest_contour_index].m10 / 
mu_dark[dark_cephalotorax_largest_contour_index].m00),
				(int)(mu_dark[dark_cephalotorax_largest_contour_index].m01 / 
mu_dark[dark_cephalotorax_largest_contour_index].m00));

// Computing circularity of the convex hull of dark part of the cephalothorax -------------------

	vector<vector<Point>> convex_hull(dark_cephalothorax_contours.size());
	for (size_t i = 0; i < dark_cephalothorax_contours.size(); i++)
	{
		convexHull(dark_cephalothorax_contours[i], convex_hull[i]);
	}

	float perimeter, area, circularity;

	perimeter = arcLength(convex_hull[dark_cephalotorax_largest_contour_index], true);
	area = contourArea(convex_hull[dark_cephalotorax_largest_contour_index]);
	circularity = 4.0 * 3.1416 * (area / (perimeter*perimeter));
	drawContours(dark_cephalothorax, convex_hull, dark_cephalotorax_largest_contour_index, 
Scalar(255, 255, 255), -1);


// Adjusting a line to the dark part of the cephalotorax -------------------

	Vec4f line;
	fitLine(dark_cephalothorax_contours[dark_cephalotorax_largest_contour_index], line, 
CV_DIST_L2, 0, 0.01, 0.01); //L2

	float normal_x, normal_y;
	normal_x = (float)centroid.x - line[2];
	normal_y = (float)centroid.y - line[3];

	float module = sqrt(normal_x*normal_x + normal_y * normal_y);
	normal_x = normal_x / module;
	normal_y = normal_y / module;

	if (circularity >= CIRCULARITY_LIMIT) {
		line[0] = normal_y;
		line[1] = -normal_x;
	}

	Point c, p1, p2;
	float A, B, C, D;
	float cx, cy;
	float D2;
```

```
	c.x = (int)line[2];
	c.y = (int)line[3];
	p1.x = (int)(line[0] * LINE_EXTENSION + line[2]);
	p1.y = (int)(line[1] * LINE_EXTENSION + line[3]);
	p2.x = (int)(line[0] * - LINE_EXTENSION + line[2]);
	p2.y = (int)(line[1] * - LINE_EXTENSION + line[3]);

	A = line[1];
	B = -line[0];
	C = line[3] * line[0] - line[2] * line[1];

	cv::line(pupae_contour, p1, p2, Scalar(150, 150, 150), 2, 8);

	cv::line(res_2, p1, p2, Scalar(150, 150, 150), 2, 8);
	circle(res_2, centroid_dark, 3, cvScalar(100, 100, 100), 3, 3, 0);

// Finding eye candidates -------------------

	Mat binary_b;
	threshold(bgr[0], binary_b, EYE_CANDIDATES_THRESHOLD, 255,
CV_THRESH_BINARY_INV);

	Mat eye_candidate = binary_b.clone();
	eye_candidate.setTo(Scalar(0, 0, 0));

	vector<vector<Point>> binary_b_contours;
	findContours(binary_b, binary_b_contours, CV_RETR_LIST,
CV_CHAIN_APPROX_NONE);

	largest_area = 0;
	int binary_b_largest_contour_index = 0;
	for (int idx = 0; idx < binary_b_contours.size(); idx++) {
		double a = contourArea(binary_b_contours[idx], false);
		if (a > largest_area) {
			largest_area = a;
			binary_b_largest_contour_index = idx;
		}
	}

	for (int idx = 0; idx < binary_b_contours.size(); idx++) {
		if (idx != binary_b_largest_contour_index) {
			drawContours(eye_candidate, binary_b_contours, idx, Scalar(255, 255, 255), -
1);
		}
	}

	Mat pupae_eroded_contour = pupae_contour_2.clone();
	erode(pupae_contour_2, pupae_eroded_contour, Mat(), Point(-1, -1), 10);
	subtract(eye_candidate, pupae_eroded_contour, eye_candidate);

// Filtering eye candidates -------------------

	Mat filtered_eyes = binary_g.clone();
	filtered_eyes.setTo(Scalar(0, 0, 0));
```

```
	vector<vector<Point>> eye_candidates_contours;
	findContours(eye_candidate, eye_candidates_contours, CV_RETR_LIST,
CV_CHAIN_APPROX_NONE);
	vector<Moments> mu2(eye_candidates_contours.size());

	for (int idx = 0; idx < eye_candidates_contours.size(); idx++) {
		double a = contourArea(eye_candidates_contours[idx], false);
		mu2[idx] = moments(eye_candidates_contours[idx], false);
		float cx, cy;
		float D2;

		if (mu2[idx].m00 != 0) {
			cx = mu2[idx].m10 / mu2[idx].m00;
			cy = mu2[idx].m01 / mu2[idx].m00;
			D = abs(A*cx + B * cy + C) / sqrt(A*A + B * B);
			D2 = sqrt(((float)centroid.x - cx)*((float)centroid.x - cx) + ((float)centroid.y -
cy)*((float)centroid.y - cy));
		}
		else {
			cx = 0;
			cy = 0;
			D = 0;
			D2 = 0;
			a = 0;
		}

		if ((D > MIN_DISTANCE_TO_LINE) && (D < MAX_DISTANCE_TO_LINE) &&
(D2<MAX_DISTANCE_TO_CENTROID) && (a > MINIMUM_EYE_SIZE)) {

			double sign, module_1, module_2;
			double ACx, ACy, ABx, ABy, ADx, ADy;

			ACx = (centroid.x - line[2]);
			ACy = (centroid.y - line[3]);
			ADx = cx - line[2];
			ADy = cy - line[3];
			ABx = line[0];
			ABy = line[1];
			module_1 = sqrt((float)(ACx * ACx + ACy * ACy));
			module_2 = sqrt((float)(ADx * ADx + ADy * ADy));
			ACx = ACx / module_1;
			ACy = ACy / module_1;
			ADx = ADx / module_2;
			ADy = ADy / module_2;

			sign = (ABx * ACy - ABy * ACx) * (ABx * ADy - ABy * ADx);
			if (sign > 0) {
				drawContours(filtered_eyes, eye_candidates_contours, idx, Scalar(255,
255, 255), -1);
			}

		}
```

```
	}

	vector<vector<Point>> eye_contours;
	findContours(filtered_eyes, eye_contours, CV_RETR_LIST,
CV_CHAIN_APPROX_NONE);

	int male = 0;
	CvPoint eye_centroid;

	if (eye_contours.size() > 0) {
		male = 1;
		largest_area = 0;
		int largest_eye_contour_index = 0;
		for (int idx = 0; idx < eye_contours.size(); idx++) {
			double a = contourArea(eye_contours[idx], false);
			if (a > largest_area) {
				largest_area = a;
				largest_eye_contour_index = idx;
			}
		}

		vector<Moments> mu3(eye_contours.size());
		for (int i = 0; i < eye_contours.size(); i++)
		{
			mu3[i] = moments(eye_contours[i], false);
		}

		eye_centroid = cvPoint((int)(mu3[largest_eye_contour_index].m10 /
mu3[largest_eye_contour_index].m00), (int)(mu3[largest_eye_contour_index].m01 /
mu3[largest_eye_contour_index].m00));

		drawContours(res_2, eye_contours, largest_eye_contour_index, Scalar(0, 0, 0), -1);
	}

	char buffer[25];

	sprintf_s(buffer, "(%d)", current_pupae++);
	putText(res_1, buffer, Point(3, 25), FONT_HERSHEY_SIMPLEX, 0.6, Scalar(0, 0, 0), 2);
	putText(res_2, buffer, Point(3, 25), FONT_HERSHEY_SIMPLEX, 0.6, Scalar(0, 0, 0), 2);
	sprintf_s(buffer, "%.2f", circularity);
	putText(res_2, buffer, Point(150, 190), FONT_HERSHEY_SIMPLEX, 0.6, Scalar(0, 0, 0),
2);

	if (male) {
		subtract(res_1, male_symbol, res_1);
		subtract(res_2, male_symbol, res_2);
		circle(res_1, eye_centroid, 15, cvScalar(0, 0, 0), 2);
	} else {
		subtract(res_1, female_symbol, res_1);
		subtract(res_2, female_symbol, res_2);
	}

	res_1.copyTo(mosaic_1(Rect(x, y, res_1.cols, res_1.rows)));
	res_2.copyTo(mosaic_2(Rect(x, y, res_2.cols, res_2.rows)));
```

```
	col++;
	if (col<10)
		x += IMAGE_SIZE;
	else
	{
		col = 0;
		x = 0;
		y += IMAGE_SIZE;
	}

}


int main()
{

	int k;
	int i = 1;
	char s[50];

	sprintf_s(s, "resources/male.bmp");
	male_symbol = imread(s, CV_LOAD_IMAGE_COLOR);
	sprintf_s(s, "resources/female.bmp");
	female_symbol = imread(s, CV_LOAD_IMAGE_COLOR);

	cout << "OpenCV version : " << CV_VERSION << endl;

	for (int i = 1; i < 51; i++) {

		sprintf_s(s, "pupae/%d.bmp", i);
		buffer_0 = imread(s, CV_LOAD_IMAGE_COLOR);
		res_1 = buffer_0.clone();
		res_2 = buffer_0.clone();
		res_2.setTo(Scalar(255, 255, 255));

		process_image();

	}

	for (int i = 0; i<11; i++) {
		line(mosaic_1, cvPoint(i * IMAGE_SIZE, 0), cvPoint(i * IMAGE_SIZE,
IMAGE_SIZE * 5), cvScalar(100, 100, 100));
		line(mosaic_2, cvPoint(i * IMAGE_SIZE, 0), cvPoint(i * IMAGE_SIZE,
IMAGE_SIZE * 5), cvScalar(100, 100, 100));
	}
	for (int i = 0; i<6; i++) {
		line(mosaic_1, cvPoint(0, i * IMAGE_SIZE), cvPoint(IMAGE_SIZE * 10, i *
IMAGE_SIZE), cvScalar(100, 100, 100));
		line(mosaic_2, cvPoint(0, i * IMAGE_SIZE), cvPoint(IMAGE_SIZE * 10, i *
IMAGE_SIZE), cvScalar(100, 100, 100));
	}

	imwrite("results/mosaic_1.jpg", mosaic_1);
	imwrite("results/mosaic_2.jpg", mosaic_2);
```

```
    return 0;
}
```
