# Supplementary material for: Genetic sexing strains for the population suppression of the mosquito vector Aedes aegypti
Source: Philos Trans R Soc Lond B Biol Sci. 2020 Dec 28;376(1818):20190808. doi: 10.1098/rstb.2019.0808 (PMC7776939; doi:10.1098/rstb.2019.0808)
Supplement: Raw data and results from the cage population suppression experiment [file rstb20190808supp4.pdf]

Supplementary material

Electronic Supplementary Material 4: Raw data and results from the cage population suppression experiment

This document contains:

1. **The stochastic model for the Red-eye GSS and the Red-eye GSS/Inv35**
    a. Comparative model figures
    b. Comparative model analysis
    c. Comparative overall analysis
2. **Quantifications**
    a. Number of eggs collected each round
    b. Number of females introduced each round
3. **Calculated parameters**
    a. Fried Index – male competitiveness
    b. Induced Sterility Index

1. Stochastic Model – Red-GSS and Red-GSS/Inv35
A new variable using random numbers was created through a uniform distribution (ranging from 0 to 1, representing hatching rates) for the same number of samples existing on the current collected dataset.
This procedure was repeated six times, using for each repetition a different seed to initiate the randomization, resulting on six different datasets for comparison.

Red-GSS Model Summary
General Formula:
Hatch rate ~ cross + release

Model #	Rank	Df.res	AIC	AICc	BIC	p.value
1	5	99	114.8	115.7	130.7	1.39E-05
2	5	99	109.9	110.8	125.8	1.19E-05
3	5	99	114.5	115.4	130.4	1.27E-05
4	5	99	108.7	109.6	124.6	4.39E-06
5	5	99	114.2	115.1	130.1	1.24E-05
6	5	99	108.3	109.2	124.2	5.64E-06

Graphical visualization comparing the random variable with the observed model:

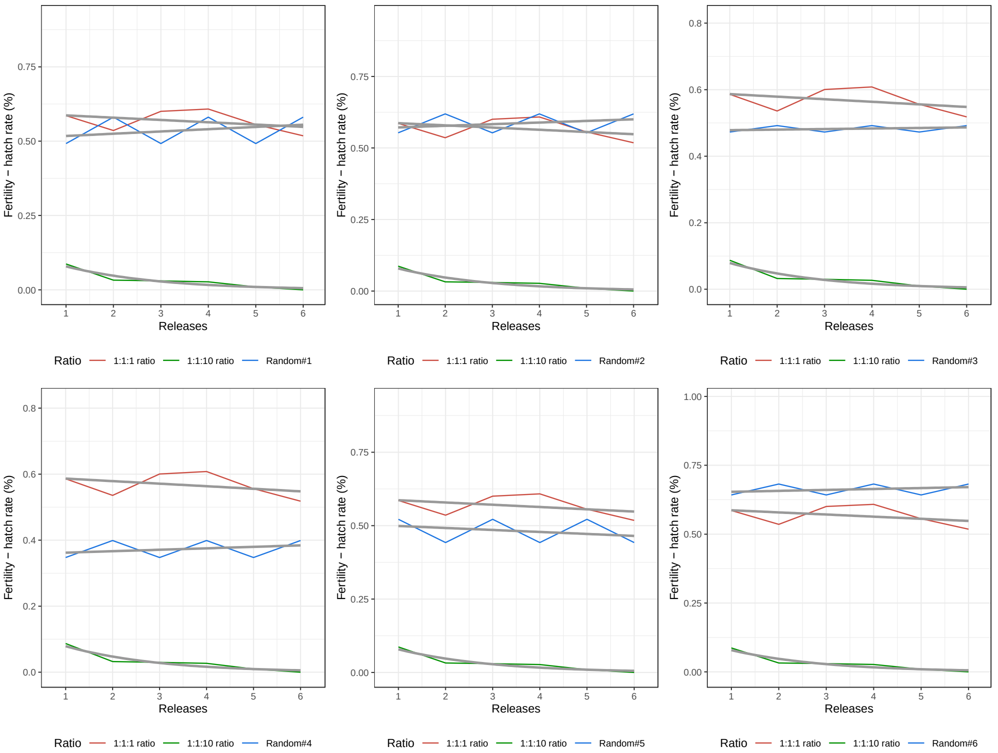

There was no major significance among the model, although each model had a different AIC and BIC value. On the other hand, all presented models had no statistical difference comparing the strains with the Random for each dataset.

Model Coefficients:		Estimate	Std. Error	z-value	Pr(>|z|)	
Random#1	(Intercept)	0.23475	0.65775	0.357	0.7212	
	1:10 ratio	-3.51737	1.4899	-2.361	0.0182	*
	Fertile CTRL	1.62086	0.84524	1.918	0.0552	.
	Random	-0.12536	0.54848	-0.229	0.8192	
	release	0.01049	0.12992	0.081	0.9356	
Random#2	(Intercept)	0.252411	0.660477	0.382	0.7023	
	1:10 ratio	-3.519786	1.489987	-2.362	0.0182	*
	Fertile CTRL	1.620793	0.84522	1.918	0.0552	.
	Random	0.07597	0.550163	0.138	0.8902	
	release	0.005441	0.130975	0.042	0.9669	
Random#3	(Intercept)	0.289978	0.65788	0.441	0.6594	
	1:10 ratio	-3.52515	1.490121	-2.366	0.018	*
	Fertile CTRL	1.620791	0.845219	1.918	0.0552	.
	Random	-0.341667	0.54819	-0.623	0.5331	
	release	-0.005292	0.129745	-0.041	0.9675	
Random#4	(Intercept)	0.264113	0.664389	0.398	0.691	
	1:10 ratio	-3.521425	1.490067	-2.363	0.0181	*
	Fertile CTRL	1.620771	0.845214	1.918	0.0552	.
	Random	-0.789494	0.552719	-1.428	0.1532	
	release	0.002097	0.132533	0.016	0.9874	
Random#5	(Intercept)	0.36846	0.65895	0.559	0.5761	
	1:10 ratio	-3.53731	1.49057	-2.373	0.0176	*
	Fertile CTRL	1.62144	0.84538	1.918	0.0551	.
	Random	-0.34339	0.54834	-0.626	0.5312	
	release	-0.02767	0.12982	-0.213	0.8312	

Random#6	(Intercept)	0.2739115	0.6692264	0.409	0.6823	
	1:10 ratio	-3.5228194	1.4901539	-2.364	0.0181	*
	Fertile CTRL	1.6207672	0.8452133	1.918	0.0552	.
	Random	0.4021965	0.556005	0.723	0.4695	
	release	-0.0007032	0.1344643	-0.005	0.9958	

Red-GSS/Inv35 Model Summary
General Formula:
Hatch rate ~ cross + release

Model #	Rank	Df.res	AIC	AICc	BIC	p.value
1	6	210	215.3	215.9	239	1.72E-11
2	6	210	214.7	215.3	238.3	1.01E-11
3	6	210	224.2	224.7	247.8	2.07E-11
4	6	210	203.1	203.6	226.7	3.56E-15
5	6	210	214.4	215	238	6.08E-13
6	6	210	181	181.6	204.7	5.23E-16

Graphical visualization comparing the random variable with the observed model:

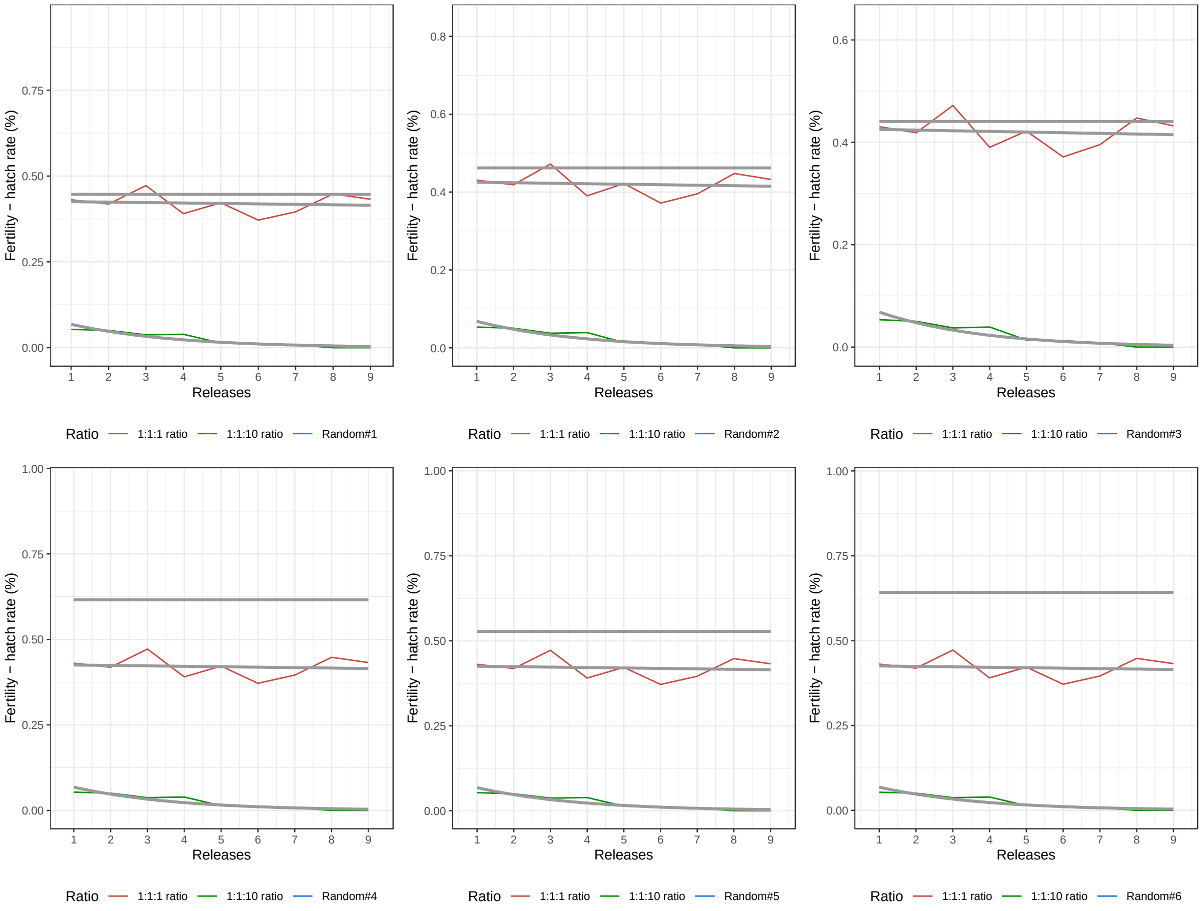

There was no major significance among the model, although each one had an AIC and BIC different, on the other hand most of presented models had no statistical difference comparing the strains with the Random for each dataset.

Model Coefficients:		Estimate	Std. Error	z-value	Pr(>|z|)	
Random#1	(Intercept)	-0.285232	0.49619	-0.575	0.5654	
	1:10 ratio	-3.390662	1.320762	-2.567	0.0103	*
	Sterile CTRL	-4.350958	2.047048	-2.125	0.0335	*
	Fertile CTRL	1.216796	0.576075	2.112	0.0347	*

	Random	0.107423	0.43533	0.247	0.8051	
	release	-0.007419	0.061469	-0.121	0.9039	
Random#2	(Intercept)	-0.285375	0.495825	-0.576	0.5649	
	1:10 ratio	-3.390661	1.320762	-2.567	0.0103	*
	Sterile CTRL	-4.350956	2.047048	-2.125	0.0335	*
	Fertile CTRL	1.216795	0.576075	2.112	0.0347	*
	Random	0.170141	0.435084	0.391	0.6958	
	release	-0.007391	0.06135	-0.12	0.9041	
Random#3	(Intercept)	-0.285168	0.496355	-0.575	0.5656	
	1:10 ratio	-3.390663	1.320762	-2.567	0.0103	*
	Sterile CTRL	-4.350958	2.047048	-2.125	0.0335	*
	Fertile CTRL	1.216796	0.576075	2.112	0.0347	*
	Random	0.084434	0.435442	0.194	0.8463	
	release	-0.007432	0.061522	-0.121	0.9038	
Random#4	(Intercept)	-0.284144	0.498962	-0.569	0.569	
	1:10 ratio	-3.390671	1.320763	-2.567	0.0103	*
	Sterile CTRL	-4.350966	2.047048	-2.125	0.0335	*
	Fertile CTRL	1.216802	0.576077	2.112	0.0347	*
	Random	0.793119	0.437236	1.814	0.0697	.
	release	-0.007637	0.062365	-0.122	0.9025	
Random#5	(Intercept)	-0.285442	0.495654	-0.576	0.5647	
	1:10 ratio	-3.390661	1.320762	-2.567	0.0103	*
	Sterile CTRL	-4.350956	2.047048	-2.125	0.0335	*
	Fertile CTRL	1.216795	0.576075	2.112	0.0347	*
	Random	0.433551	0.434969	0.997	0.3189	
	release	-0.007377	0.061295	-0.12	0.9042	
Random#6	(Intercept)	-0.283391	0.500872	-0.566	0.5715	
	1:10 ratio	-3.390677	1.320763	-2.567	0.0103	*
	Sterile CTRL	-4.350973	2.047049	-2.125	0.0335	*
	Fertile CTRL	1.216807	0.576078	2.112	0.0347	*
	Random	0.908093	0.438583	2.071	0.0384	*
	release	-0.007788	0.062979	-0.124	0.9016	

2. Raw data from the different releases of the cage suppression experiment

strain	release	cross	replicate	n_females	n_eggs	n_larvae	eggs_f	hatch_rate
red-gss	1	CTRL	A	125	1077	959	8,6	0,890
red-gss	1	CTRL	B	125	1018	903	8,1	0,887
red-gss	1	CTRL	C	125	1075	944	8,6	0,878
red-gss	1	1_1	A	83	1070	641	12,9	0,599
red-gss	1	1_1	B	83	1052	651	12,7	0,619
red-gss	1	1_1	C	83	1052	568	12,7	0,540
red-gss	1	1_10	A	20	364	33	18,2	0,091
red-gss	1	1_10	B	20	377	50	18,9	0,133
red-gss	1	1_10	C	20	395	15	19,8	0,038
red-gss	2	CTRL	A	83	1070	838	12,9	0,783
red-gss	2	CTRL	B	83	1052	848	12,7	0,806
red-gss	2	CTRL	C	82	1052	865	12,8	0,822
red-gss	2	1_1	A	62	1062	590	17,1	0,556
red-gss	2	1_1	B	65	1065	545	16,4	0,512
red-gss	2	1_1	C	57	1031	557	18,1	0,540
red-gss	2	1_10	A	9	373	17	41,4	0,046
red-gss	2	1_10	B	14	393	16	28,1	0,041
red-gss	2	1_10	C	4	183	2	45,8	0,011
red-gss	3	CTRL	A	73	1184	1023	16,2	0,864
red-gss	3	CTRL	B	75	1106	1008	14,7	0,911
red-gss	3	CTRL	C	77	1188	1003	15,4	0,844
red-gss	3	1_1	A	66	971	576	14,7	0,593
red-gss	3	1_1	B	59	874	565	14,8	0,646
red-gss	3	1_1	C	61	945	531	15,5	0,562
red-gss	3	1_10	A	7	329	17	47,0	0,052
red-gss	3	1_10	B	6	364	14	60,7	0,038
red-gss	3	1_10	C	2	84	0	42,0	0,000
red-gss	4	CTRL	A	81	1007	852	12,4	0,846
red-gss	4	CTRL	B	85	998	885	11,7	0,887
red-gss	4	CTRL	C	79	827	761	10,5	0,920
red-gss	4	1_1	A	64	932	558	14,6	0,599
red-gss	4	1_1	B	66	951	559	14,4	0,588
red-gss	4	1_1	C	62	840	536	13,5	0,638
red-gss	4	1_10	A	5	325	10	65,0	0,031
red-gss	4	1_10	B	3	130	3	43,3	0,023
red-gss	4	1_10	C	0	0	0		
red-gss	5	CTRL	A	79	1146	1057	14,5	0,922
red-gss	5	CTRL	B	83	1099	885	13,2	0,805
red-gss	5	CTRL	C	86	1172	1091	13,6	0,931
red-gss	5	1_1	A	67	832	489	12,4	0,588
red-gss	5	1_1	B	62	965	498	15,6	0,516
red-gss	5	1_1	C	65	989	558	15,2	0,564
red-gss	5	1_10	A	3	102	2	34,0	0,020
red-gss	5	1_10	B	2	50	0	25,0	0,000
red-gss	5	1_10	C	0	0	0		

red-gss	6	CTRL	A	86	1034	922	12,0	0,892
red-gss	6	CTRL	B	75	989	885	13,2	0,895
red-gss	6	CTRL	C	87	1128	967	13,0	0,857
red-gss	6	1_1	A	59	858	490	14,5	0,571
red-gss	6	1_1	B	60	781	380	13,0	0,487
red-gss	6	1_1	C	56	805	400	14,4	0,497
red-gss	6	1_10	A	1	86	0	86,0	0,000
red-gss	6	1_10	B	0	0	0		
red-gss	6	1_10	C	0	0	0		
red-gss-i35	1	CTRL WT	A	125	3784	2775	30,3	0,733
red-gss-i35	1	CTRL WT	B	125	4320	3109	34,6	0,720
red-gss-i35	1	CTRL WT	C	125	4043	3112	32,3	0,770
red-gss-i35	1	CTRL ST	A	125	3829	37	30,6	0,010
red-gss-i35	1	CTRL ST	B	125	3820	55	30,6	0,014
red-gss-i35	1	CTRL ST	C	125	3962	26	31,7	0,007
red-gss-i35	1	1_1	A	83	2707	1190,0	32,6	0,440
red-gss-i35	1	1_1	B	83	2808	1292,0	33,8	0,460
red-gss-i35	1	1_1	C	83	2595	1018,0	31,3	0,392
red-gss-i35	1	1_10	A	20	648	34	32,4	0,052
red-gss-i35	1	1_10	B	20	676	33	33,8	0,049
red-gss-i35	1	1_10	C	20	612	36	30,6	0,059
red-gss-i35	2	CTRL WT	A	68	3961	2863	58,3	0,723
red-gss-i35	2	CTRL WT	B	67	3898	2692	58,2	0,691
red-gss-i35	2	CTRL WT	C	72	4083	2629	56,7	0,644
red-gss-i35	2	CTRL ST	A	68	3928	52	57,8	0,013
red-gss-i35	2	CTRL ST	B	67	3972	38	59,3	0,010
red-gss-i35	2	CTRL ST	C	72	4110	28	57,1	0,007
red-gss-i35	2	1_1	A	56	3159	1308,0	56,4	0,414
red-gss-i35	2	1_1	B	59	3098	1180,0	52,5	0,381
red-gss-i35	2	1_1	C	47	3082	1421,0	65,6	0,461
red-gss-i35	2	1_10	A	6	556	28	92,7	0,050
red-gss-i35	2	1_10	B	6	483	26	80,5	0,054
red-gss-i35	2	1_10	C	7	511	24	73,0	0,047
red-gss-i35	3	CTRL WT	A	67	4293	3268	64,1	0,761
red-gss-i35	3	CTRL WT	B	64	4147	2797	64,8	0,674
red-gss-i35	3	CTRL WT	C	60	4253	2841	70,9	0,668
red-gss-i35	3	CTRL ST	A	67	4138	59	61,8	0,014
red-gss-i35	3	CTRL ST	B	64	3905	38	61,0	0,010
red-gss-i35	3	CTRL ST	C	60	4264	32	71,1	0,008
red-gss-i35	3	1_1	A	53	3344	1551,0	63,1	0,464
red-gss-i35	3	1_1	B	51	3235	1661,0	63,4	0,513
red-gss-i35	3	1_1	C	67	3814	1673,0	56,9	0,439
red-gss-i35	3	1_10	A	6	423	16	70,5	0,038
red-gss-i35	3	1_10	B	7	473	17	67,6	0,036
red-gss-i35	3	1_10	C	7	472	18	67,4	0,038
red-gss-i35	4	CTRL WT	A	71	4469	3060	62,9	0,685
red-gss-i35	4	CTRL WT	B	63	4206	2988	66,8	0,710
red-gss-i35	4	CTRL WT	C	62	4165	2998	67,2	0,720

red-gss-i35	4	CTRL ST	A	71	4125	59	58,1	0,014
red-gss-i35	4	CTRL ST	B	63	4451	25	70,7	0,006
red-gss-i35	4	CTRL ST	C	62	3802	36	61,3	0,009
red-gss-i35	4	1_1	A	57	3346	1440	58,7	0,430
red-gss-i35	4	1_1	B	71	3746	1378	52,8	0,368
red-gss-i35	4	1_1	C	61	3798	1416	62,3	0,373
red-gss-i35	4	1_10	A	4	371	14	92,8	0,038
red-gss-i35	4	1_10	B	5	381	15	76,2	0,039
red-gss-i35	4	1_10	C	6	425	17	70,8	0,040
red-gss-i35	5	CTRL WT	A	64	4062	2970	63,5	0,731
red-gss-i35	5	CTRL WT	B	66	4145	2986	62,8	0,720
red-gss-i35	5	CTRL WT	C	67	3956	2955	59,0	0,747
red-gss-i35	5	CTRL ST	A	64	4012	27	62,7	0,007
red-gss-i35	5	CTRL ST	B	66	3927	45	59,5	0,011
red-gss-i35	5	CTRL ST	C	67	3942	32	58,8	0,008
red-gss-i35	5	1_1	A	59	3728	1643	63,2	0,441
red-gss-i35	5	1_1	B	48	3673	1576	76,5	0,429
red-gss-i35	5	1_1	C	48	3490	1383	72,7	0,396
red-gss-i35	5	1_10	A	5	365	6	73,0	0,016
red-gss-i35	5	1_10	B	5	352	5	70,4	0,014
red-gss-i35	5	1_10	C	6	392	4	65,3	0,010
red-gss-i35	6	CTRL WT	A	68	4197	2938	61,7	0,700
red-gss-i35	6	CTRL WT	B	67	4402	3119	65,7	0,709
red-gss-i35	6	CTRL WT	C	70	4339	3233	62,0	0,745
red-gss-i35	6	CTRL ST	A	68	4242	36	62,4	0,008
red-gss-i35	6	CTRL ST	B	67	3967	48	59,2	0,012
red-gss-i35	6	CTRL ST	C	70	4086	44	58,4	0,011
red-gss-i35	6	1_1	A	56	3401	1195	60,7	0,351
red-gss-i35	6	1_1	B	55	3660	1360	66,5	0,372
red-gss-i35	6	1_1	C	49	3229	1265	65,9	0,392
red-gss-i35	6	1_10	A	2	241	3	120,5	0,012
red-gss-i35	6	1_10	B	1	210	2	210,0	0,010
red-gss-i35	6	1_10	C	2	265	4	132,5	0,015
red-gss-i35	7	CTRL WT	A	65	4239	2984	65,2	0,704
red-gss-i35	7	CTRL WT	B	66	4045	2868	61,3	0,709
red-gss-i35	7	CTRL WT	C	70	4199	2950	60,0	0,703
red-gss-i35	7	CTRL ST	A	65	3998	35	61,5	0,009
red-gss-i35	7	CTRL ST	B	66	4001	37	60,6	0,009
red-gss-i35	7	CTRL ST	C	70	4351	47	62,2	0,011
red-gss-i35	7	1_1	A	47	3154	1204	67,1	0,382
red-gss-i35	7	1_1	B	49	3323	1339	67,8	0,403
red-gss-i35	7	1_1	C	49	3218	1295	65,7	0,402
red-gss-i35	7	1_10	A	1	142	1	142,0	0,007
red-gss-i35	7	1_10	B	1	135	1	135,0	0,007
red-gss-i35	7	1_10	C	2	187	2	93,5	0,011
red-gss-i35	8	CTRL WT	A	66	4095	2955	62,0	0,722
red-gss-i35	8	CTRL WT	B	66	4082	2820	61,8	0,691
red-gss-i35	8	CTRL WT	C	66	4229	2965	64,1	0,701

red-gss-i35	8	CTRL ST	A	66	4040	21	61,2	0,005
red-gss-i35	8	CTRL ST	B	66	4047	22	61,3	0,005
red-gss-i35	8	CTRL ST	C	66	4133	26	62,6	0,006
red-gss-i35	8	1_1	A	50	3192	1465,0	63,8	0,459
red-gss-i35	8	1_1	B	53	3667	1604,0	69,2	0,437
red-gss-i35	8	1_1	C	53	3594	1606,0	67,8	0,447
red-gss-i35	8	1_10	A	0	32	0		0,000
red-gss-i35	8	1_10	B	0	48	0		0,000
red-gss-i35	8	1_10	C	2	46	0	23,0	0,000
red-gss-i35	9	CTRL WT	A	67	4095	2855	61,1	0,697
red-gss-i35	9	CTRL WT	B	64	4082	2730	63,8	0,669
red-gss-i35	9	CTRL WT	C	65	4029	2895	62,0	0,719
red-gss-i35	9	CTRL ST	A	67	3940	37	58,8	0,009
red-gss-i35	9	CTRL ST	B	64	3447	22	53,9	0,006
red-gss-i35	9	CTRL ST	C	65	3733	36	57,4	0,010
red-gss-i35	9	1_1	A	60	3692	1575,0	61,5	0,427
red-gss-i35	9	1_1	B	59	3567	1584,0	60,5	0,444
red-gss-i35	9	1_1	C	60	3394	1447,0	56,6	0,426
red-gss-i35	9	1_10	A	0	16	0		0,000
red-gss-i35	9	1_10	B	0	12	0		0,000
red-gss-i35	9	1_10	C	0	28	0		0,000

3. Induced Sterility across releases

Strain	Release	ISI	mean ISI
Red-eye GSS 1:1	1	0.34	0.35
	2	0.33	
	3	0.31	
	4	0.31	
	5	0.38	
	6	0.41	
Red-eye GSS-Inv35 1:1	1	0.42	0.41
	2	0.39	
	3	0.33	
	4	0.45	
	5	0.42	
	6	0.48	
	7	0.44	
	8	0.37	
	9	0.38	

Strain	Release	ISI	mean ISI
Red-eye GSS 1:10	1	0.90	0.96
	2	0.95	
	3	0.95	
	4	0.97	
	5	0.99	
	6	1.00	
Red-eye GSS-Inv35 1:10	1	0.93	0.97
	2	0.93	
	3	0.95	
	4	0.94	
	5	0.98	
	6	0.98	
	7	0.99	
	8	1.00	
	9	1.00	

[2]Induced Sterility
